# Multiscale model for forecasting Sabin 2 vaccine virus household and community transmission

**DOI:** 10.1371/journal.pcbi.1009690

**Published:** 2021-12-21

**Authors:** Michael Famulare, Wesley Wong, Rashidul Haque, James A. Platts-Mills, Parimalendu Saha, Asma B. Aziz, Tahmina Ahmed, Md Ohedul Islam, Md Jashim Uddin, Ananda S. Bandyopadhyay, Mohammed Yunus, Khalequ Zaman, Mami Taniuchi

**Affiliations:** 1 Institute for Disease Modeling, Global Good, Intellectual Ventures, Bellevue, Washington, United States of America; 2 International Centre for Diarrhoeal Disease Research, Dhaka, Bangladesh; 3 Department of Medicine, Division of Infectious Diseases and International Health, University of Virginia, Charlottesville, Virginia, United States of America; 4 Bill & Melinda Gates Foundation, Seattle, Washington, United States of America; 5 Department of Biomedical Engineering, University of Virginia, Charlottesville, Virginia, United States of America; 6 Department of Engineering Systems and Environment, University of Virginia, Charlottesville, Virginia, United States of America; University of Tennessee Knoxville, UNITED STATES

## Abstract

Since the global withdrawal of Sabin 2 oral poliovirus vaccine (OPV) from routine immunization, the Global Polio Eradication Initiative (GPEI) has reported multiple circulating vaccine-derived poliovirus type 2 (cVDPV2) outbreaks. Here, we generated an agent-based, mechanistic model designed to assess OPV-related vaccine virus transmission risk in populations with heterogeneous immunity, demography, and social mixing patterns. To showcase the utility of our model, we present a simulation of mOPV2-related Sabin 2 transmission in rural Matlab, Bangladesh based on stool samples collected from infants and their household contacts during an mOPV2 clinical trial. Sabin 2 transmission following the mOPV2 clinical trial was replicated by specifying multiple, heterogeneous contact rates based on household and community membership. Once calibrated, the model generated Matlab-specific insights regarding poliovirus transmission following an accidental point importation or mass vaccination event. We also show that assuming homogeneous contact rates (mass action), as is common of poliovirus forecast models, does not accurately represent the clinical trial and risks overestimating forecasted poliovirus outbreak probability. Our study identifies household and community structure as an important source of transmission heterogeneity when assessing OPV-related transmission risk and provides a calibratable framework for expanding these analyses to other populations.

**Trial Registration:** ClinicalTrials.gov This trial is registered with clinicaltrials.gov, NCT02477046.

## Author summary

The oral poliovirus vaccines (OPV) have been one of the most effective tools for poliovirus eradication. These vaccines contain transmissible, live-attenuated viruses that promote mucosal immunity in the primary vaccine recipient and their nearby contacts. Although wild poliovirus is on the verge of eradication, circulating, vaccine-derived poliovirus (cVDPV) outbreaks are undermining eradication efforts and are an increasingly urgent public health issue. Models capable of accurately forecasting future outbreak risk and transmission will be crucial for informing strategies aimed at the eradication of both wild poliovirus and cVDPV. Here, we generated and calibrated a poliovirus transmission model that combines multigenerational household and community structure, intrahost infection, and immunity modeling to shedding data collected during a monovalent Sabin 2 OPV (mOPV2) clinical trial performed in Matlab, Bangladesh. This model provided a unique opportunity to determine whether the additional complexities imposed by household and community structure were necessary for accurate transmission modeling. Our study shows that household and community structure plays an important role in limiting poliovirus transmission and that its inclusion in transmission model is necessary for accurate outbreak risk prediction.

## Introduction

Mass immunization with the live-attenuated oral poliovirus vaccines (OPV) have successfully reduced wild poliovirus (WPV) incidence by 99% and resulted in the eradication of two of the three poliovirus serotypes (Type 1 and Type 2) [1–3]. However, complete poliovirus eradication must include the three OPV vaccine viruses (Sabin 1, 2, and 3), which are transmissible and capable of reverting attenuation. Circulating, vaccine-derived poliovirus (cVDPV) can cause clinical poliomyelitis cases indistinguishable from those of WPV [4,5] and is a rapidly growing public health threat. Despite the withdrawal of Sabin 2 OPV from global routine immunization schedules in 2016 (during an event known as “the Switch”), cVDPV2 (Sabin 2 cVDPV) outbreaks have been increasing in frequency. The GPEI (Global Polio Eradication Initiative) is caught in a paradoxical situation because the current cVDPV2 containment strategy relies on immunization with monovalent Sabin 2 OPV (mOPV2) [6,7]. Nearly half of the outbreaks reported in 2019 were traced to a previous mOPV2 vaccination campaign [8].

Policymakers must develop strategies that ensure the complete eradication of poliovirus even as virus is reintroduced from mOPV2 vaccination. As global immunity against Type 2 poliovirus declines, population-specific differences in demography and social mixing will be increasingly relevant for quantifying vaccine virus transmission risk and outbreak probability [9–11]. Quantities such as outbreak probability and the probability of establishing endemic transmission [12,13] are sensitive to variations in transmission structure that are not always represented in poliovirus forecast models due to their reliance on mass action, which assumes homogeneous transmission and social mixing [9,14,15]. Of the forecast models used during the 2014–2015 West African Ebola outbreak, among the most accurate was a mechanistic model that defined spatio-temporal trends in transmission data based on collected field data [16]. Adopting a similar approach and generating a poliovirus transmission model that uses transmission data collected from mOPV2 campaigns or during a cVDPV2 outbreak could improve model accuracy and enable poliovirus eradication.

Here, we present an agent-based, mechanistic model designed to assess OPV-related vaccine virus transmission risk in realistic populations with different immunity, demography, and social mixing patterns. Individual immunity and infections are simulated using a previously described poliovirus infection model informed by decades of clinical trial research [17]. The model uses a household evolution model informed by cultural anthropology studies and demographic health surveys to generate multiscale populations that organize individuals into dynamically changing households and other community structures. Simulated transmission incorporates transmission heterogeneity from differences in immunity, shedding duration, shedding concentration, shifting household compositions, and social contact preference. OPV genetic reversion [5] was not simulated in this study and is instead addressed in a follow-up study [18]. Here, we focused on comparing the transmission of a hypothetical, genetically stable Sabin 2 vaccine strain with a fully reverted cVDPV.

To showcase the utility of our model, we present a simulation of mOPV2-related Sabin 2 transmission in rural Matlab, Bangladesh based on the viral shedding data collected from infants and their household contacts during an mOPV2 clinical trial [19]. Matlab is a highly structured population whose living arrangements and household and community structure are defined by predominantly family households, *baris* (a homestead of interrelated households) and villages. Once calibrated, the model generated Matlab-specific insights regarding poliovirus transmission following an accidental point importation or mass vaccination event. We also contrast the results with a simpler mass action model that assumes homogenous mixing to identify whether social mixing patterns, such as those imposed by household and community structure, impact poliovirus forecasting.

## Materials and methods

### Ethics statement

This clinical trial was done according to the guidelines of the Declaration of Helsinki. The protocol was approved by the Research Review Committee (RRC) and Ethical Review Committee (ERC) of the International Centre for Diarrhoeal Disease Research, Bangladesh (icddr, b) and the Institutional Review Board of the University of Virginia. The data used in this project is from a randomized vaccine trial conducted in Matlab, Bangladesh in 2015–2016. Formal written consent was obtained from all adult study participants and all parent/guardian of underaged children in the study and formal verbal consent was obtained from all parent/guardian of children under 5 years of age who received the mOPV2 vaccine during the special immunization campaign. The clinical trial was registered at ClinicalTrials.gov, number NCT02477046.

### Clinical trial design and sample collection

We previously performed an mOPV2 challenge campaign in the Maternal, Child, and Family Planning intervention region of rural Matlab, Bangladesh that is described in detail in ref. [19]. A schematic of the trial design is presented in **S1 Fig**. Briefly, villages were assigned with routine immunization with trivalent OPV (Sabin 1, 2, and 3), bivalent OPV (Sabin 1 and 3) + one inactivated poliovirus (IPV) dose, or bOPV + two IPV doses. All infants were vaccinated by one of these three routine immunization schedules unless a medical contraindication was present. A subset of infants, their two youngest household contacts, and community participants less than five years of age were enrolled in the study. For the subset of infants enrolled in the study, stool samples were collected before routine immunization and at age 18 weeks. Stool samples were also collected from their household contacts when the infant was 18 weeks of age.

The mOPV2 campaign challenged approximately 33% of the enrolled infants, 6% of the household contacts, and 40% of the community participants with mOPV2. Stool samples from infants and household contacts were collected at weekly intervals in the first ten weeks following the mOPV2 vaccination campaign and at weeks 14, 18, and 22. No stool samples were collected from the community participants. For all participants, residence *bari* was known but no additional information regarding household structure (size or composition) was recorded. The stool samples collected after mOPV2 challenge were organized into a series of eight cohorts defined by the individual providing the sample (infant or household contact), their mOPV2 challenge status, and their household/*bari* membership (**Table 1**).

**Table 1:** Cohort definitions.

Cohort Number	Individual Type	Challenged with mOPV2	Infant Status	*Bari* Status	Infection Source
1	infant	+		+	Vaccination
2	infant	-		+	Household/Bari
3	infant	-		-	Village/Region
4	Household contact	+	+	+	Vaccination
5	Household contact	-	-	+	Household/Bari
6	Household contact	-	+	+	Household/Bari
7	Household contact	+	-	+	Vaccination
8	Household contact	-	-	-	Village/Region

Cohorts 1–3 are infants while cohorts 4–8 are household contacts. The “challenged with mOPV2 “column indicates whether the individuals in this cohort received Sabin 2 vaccine (+ for yes,—for no) during the mOPV2 campaign. For cohorts 4–8, the “Infant status” column indicates whether the infant of the household contact received mOPV2. The “*Bari* status” column indicates whether any member in the *bari* received mOPV2. The “Infection Source” column indicates the type of transmission each cohort is most sensitive to. Note that this column lists the most likely transmission source but that transmission from other household community members is possible. Individuals in cohorts one, four, and seven received mOPV2 and shedding in these cohorts largely reflect individual infection dynamics.

### Model structure

**Fig 1:**
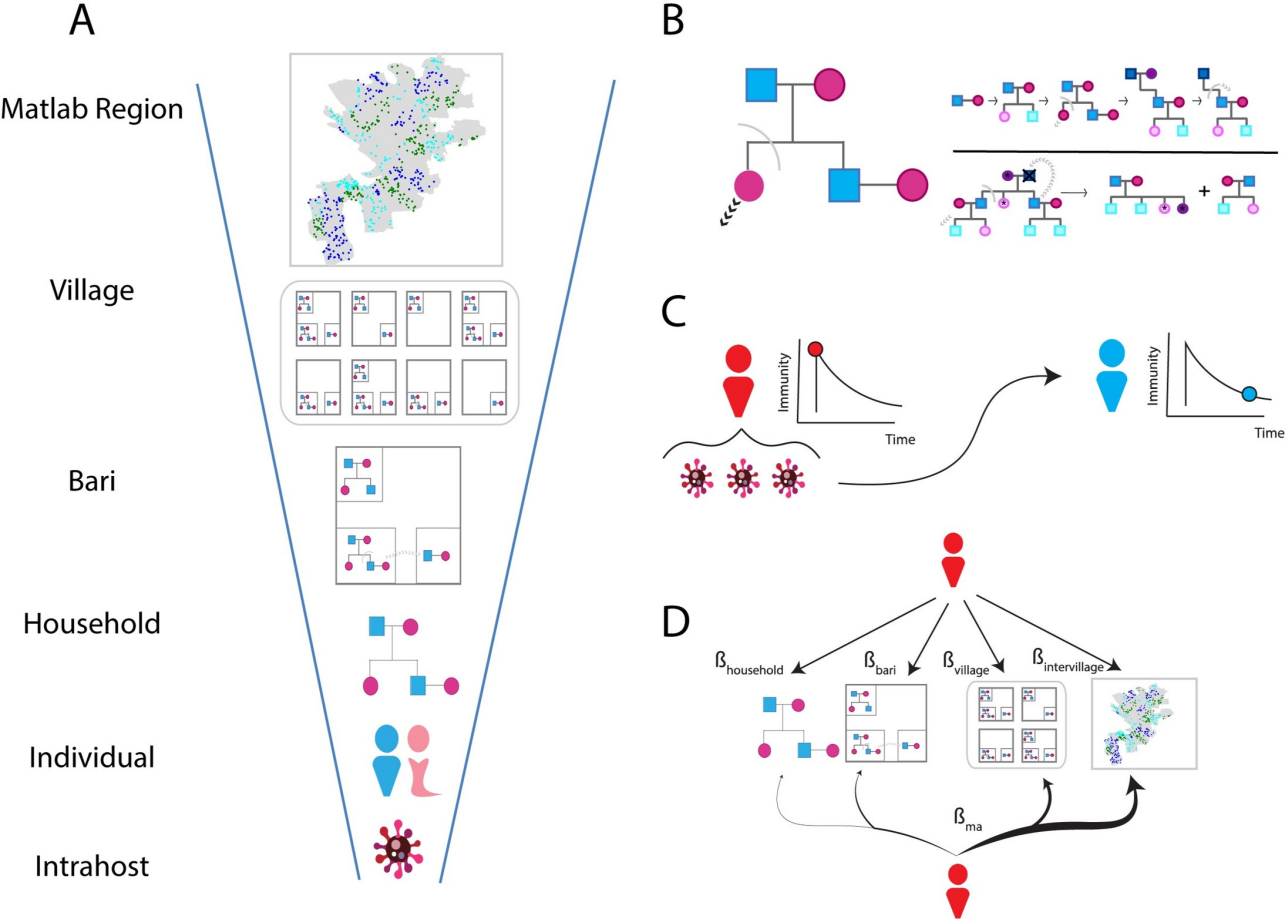
Model structure. **A)** Hierarchical demographic structure used in the model. Infected individuals are grouped into households, which are grouped into *baris*, which are grouped into villages that together define the Matlab Region. Map borders were obtained and used with permission from the International Centre for Diarrhoeal Disease Research, Bangladesh (icddr, b) (https: //github.com/InstituteforDiseaseModeling/community-structure-mediates-polio-transmission/tree/main/PopSim/data/shapefiles). **B**) Household evolution model. Households are represented as pedigree trees and their composition changes as individuals are born and removed either through death or marriage. The model is parameterized by the age- and sex-specific fertility, mortality, marriage of individuals and the rate with which newlywed couples move out after marriage (**S1 Text**). **C**) Poliovirus infection model. On infection, infected individuals receive a boost in OPV-equivalent antibody titer and assigned a shedding duration based on their titer prior to infection. Individual antibody titers are dynamic and declines over time. Viral shedding is measured in cell culture infectivity dose 50 (CCID50) units per fecal oral dose and depends on the antibody titer preceding infection and the time since the infection started. Infections shed less virus during the later stages of infection. **D**) Contact-based transmission. Simulated poliovirus transmission is the result of direct contact with infected individuals. Multiscale transmission is specified using four contact rates (β_household_, β_bari,_ β_village_, β_intervillage_) that specify the number of household, *bari*, village, and non-village members infected individuals contact per day. Homogeneous transmission (mass action) is specified with a single contact rate (β_ma_) can be used to specify the number of individuals infected individuals contact per day, regardless of household and community structure. This results in a heavy bias towards contacting non-village members who are much more numerous than thehousehold, *bari*, and village contacts of an individual.

**Fig 2:**
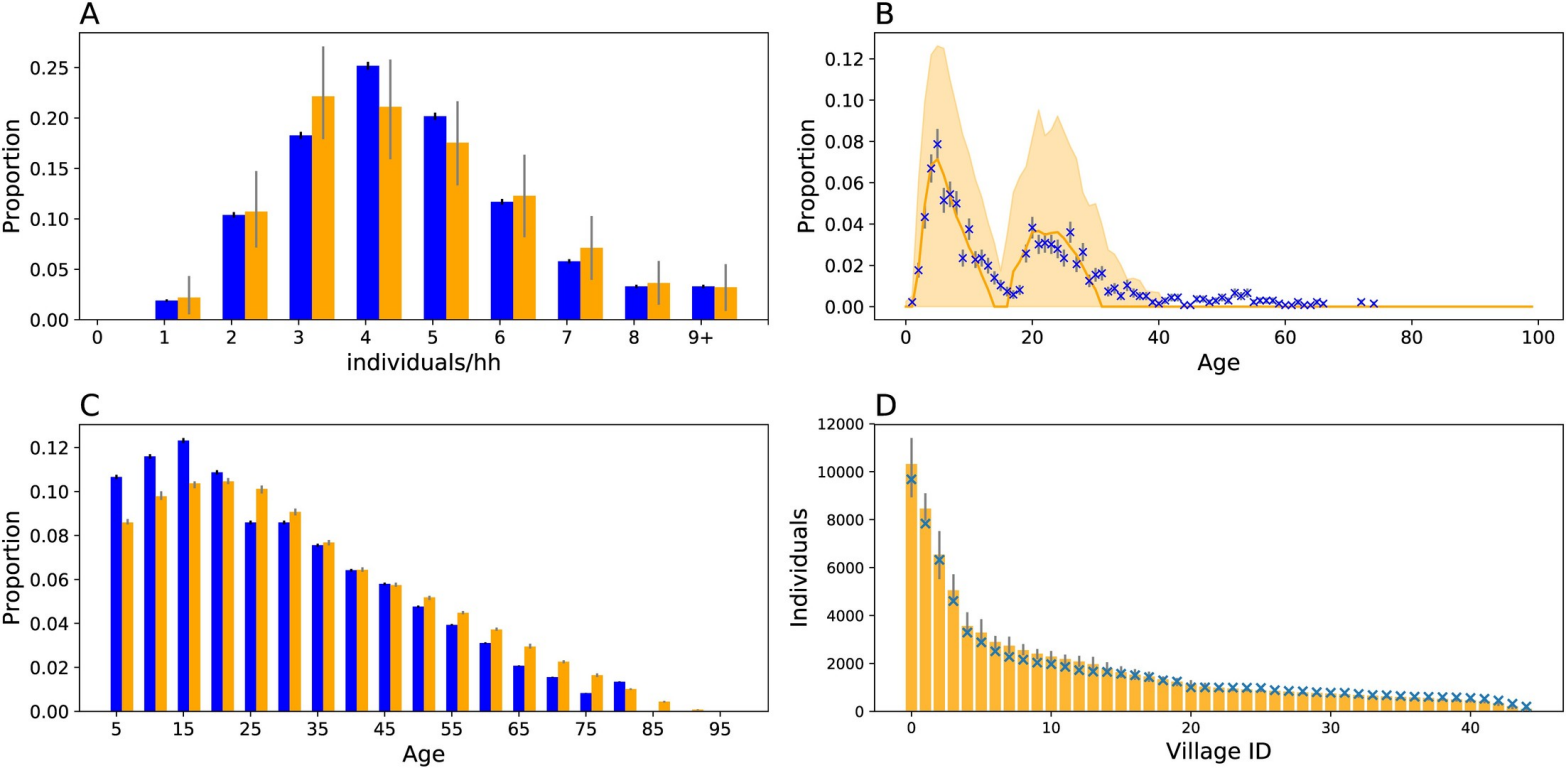
Simulating household structure. Household demography simulations (*orange*) compared against demography data from the 2014 BDHSS (blue). Error bars for simulation output represent middle 95 percentile as estimated from 20 random iterations. Error bars for the BDHSS or clinical trial data represent the 95% binomial confidence interval around the mean. A) Household size distribution. B) Housed contact age distributions. C) Age pyramid D) Village size. For D, only simulated confidence intervals are shown.

**Fig 3:**
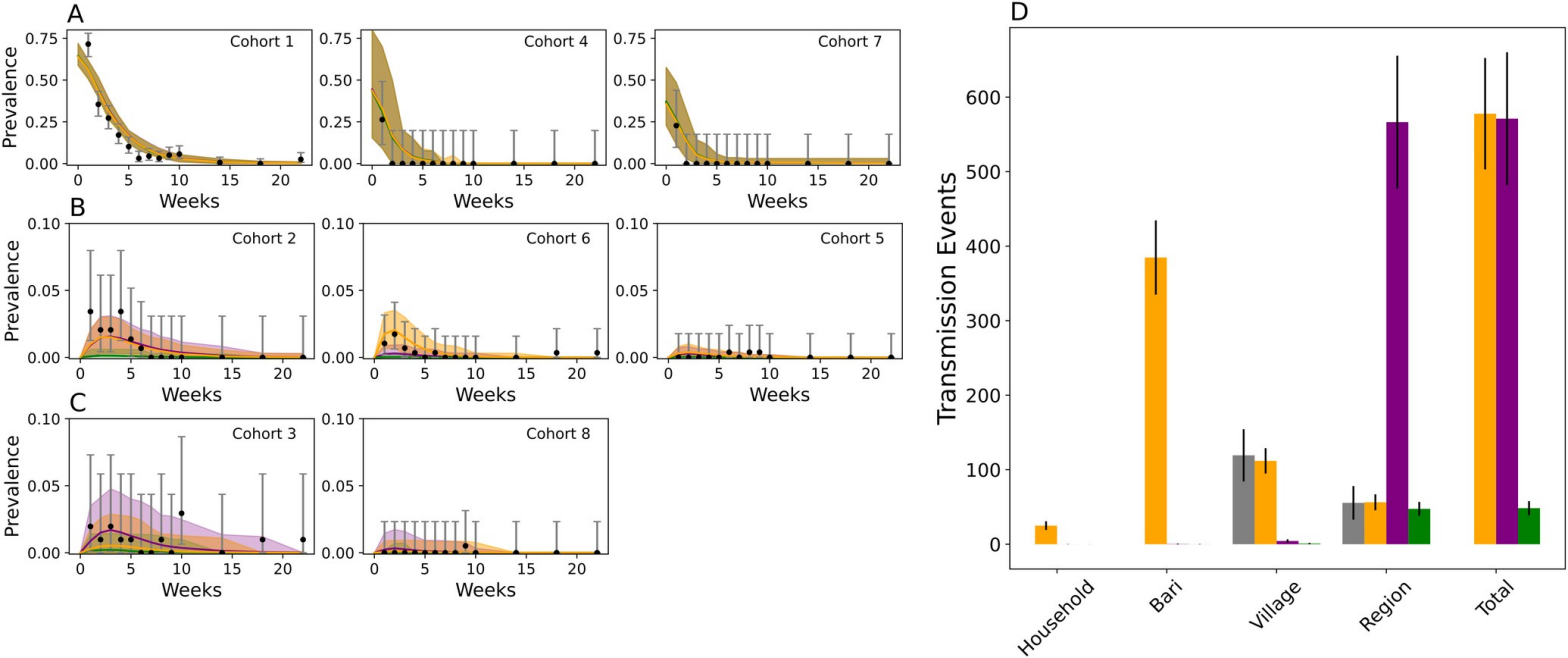
Modeled vs observed patterns of Sabin 2 following the mOPV2 vaccination campaign. **A-C**) Sabin 2 shedding in the eight infant and household contact cohorts after the mOPV2 challenge. Only data from the villages assigned with bOPV routine immunization was examined. The cohorts in **A** (left to right: cohorts one, four, seven) reflect direct vaccination with mOPV2. The cohorts in **B** (left to right: cohorts two, six, five) reflect transmission that most likely originated from a household or bari member. The cohorts in **C** transmission (left to right: cohorts three and eight) reflect transmission that most likely originated from a village or non-village member. The data and the 95% binomial confidence interval are shown in black. Simulated results with the multiscale (*orange*) and the two mass action models (*purple* and *green*) are shown. The multiscale model was calibrated to all the clinical trial data (the cohort shedding data and the tOPV data). In purple are the results from the mass action model after calibrating to the cohort shedding data alone, and in green after calibrating to all the clinical trial data. The solid line represents the simulated average and the shading indicates two standard deviations from the mean obtained from 200 simulation replicates. **D)** Distribution of transmission events in the multiscale (orange) and mass action (green) model compared against the estimates of village and non-village transmission extrapolated from tOPV-derived Sabin 2 transmission during the clinical trial (*grey*). Error bars represent the 95% confidence interval.

The model is agent-based and divided into three sub-models (**Fig 1**): 1) a household evolution model that simulates changes in composition due to births, deaths, and marriage (**Fig 1B**), 2) a previously described poliovirus infection model [17] that determines susceptibility, shedding duration, and viral shedding concentration (**Fig 1C**), and 3) a contact-based transmission model that mediates transmission by specifying the rate with which infected individuals contact other individuals based on their respective household and community memberships (**Fig 1D**). A brief description of each model is presented below and further details are reported in the **S1 Text**.

The poliovirus infection model defines immunity as the OPV-equivalent antibody titer and models susceptibility to infection as a dose-dependent response [17] (**S1 Text**). A consequence of this model is that individuals have a non-zero probability of being infected for all levels of pre-exposure immunity, with the probability of infection depending on the degree of immunity and viral dose. OPV-equivalent antibody is a measurement of the serum neutralizing antibody titers and peaks immediately after infection and wane over time. Individuals with lower OPV-equivalent antibody titers prior to infection shed more virus and for longer periods of time than individuals with higher OPV-equivalent antibody titers.

Households and demographic structure are generated using the household evolution model. The model generates multigenerational families by simulating household evolution based on anthropological frameworks of household formation and dissociation [20,21] and fertility, mortality, and marriage statistics from demographic health survey reports [22–24]. This model was used to generate multiscale populations where individuals and transmission events are grouped into a series of nested demographic scales that define household and community structure. For Matlab, these scales were defined by households, *baris*, and villages.

Transmission depends on the total viral exposure per contact and the contact structure used to specify social mixing. For each infection, the model calculates the viral exposure per contact based on the viral shedding concentration of the infected individual and the expected fecal-oral dose (grams) per contact. The average daily fecal-oral dose for Matlab was unknown and its value was determined during model calibration (**S1 Text**). During each daily timestep, infected individuals randomly sample and transmit virus to other individuals in the population. When simulating multiscale, household and community transmission, the contact-based transmission model was parameterized using four contact rates (β_household_, β_bari_, β_village_, and β_intervillage_). These rates determine the number of individuals to household, *bari*, village, and non-village members to sample. When assuming homogeneous transmission, a single contact rate, β_ma_ (where *ma* stands for mass action) determines the number of individuals to sample from the entire simulation.

## Results

### Simulating Matlab community structure

**Fig 4:**
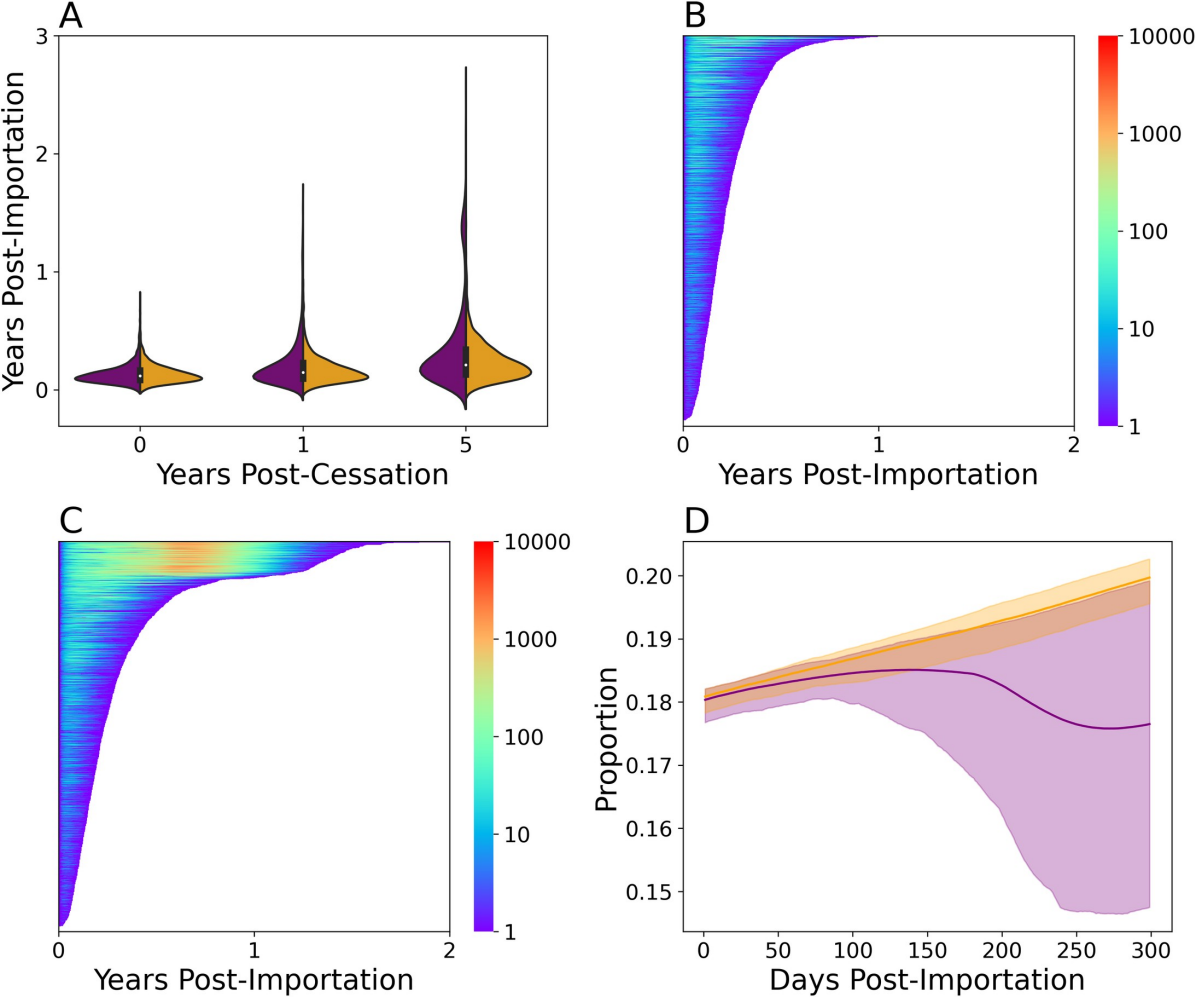
Extrapolating Sabin 2 vaccine virus transmission risk. **A**) Transmission persistence following a Sabin 2 point importation event before (year 0), one, and five years after the Switch from 2000 simulations. Simulated results with the multiscale (*orange*) and mass action (*purple*) models are shown. This version of the mass action model was calibrated to the cohort shedding data alone (**Fig 3**, *purple*). Lasagna plot of showing the total persistence times and number of infected individuals following a point importation five years after the Switch using the multiscale (**B**) and mass action (**C**) models. Each line in the lasagna plot is a different simulation and 2000 iterations are shown. The length of each line indicates the persistence time and the color the number of shedding individuals at that timepoint. **D**) Immune profile of the first 300 days after a point importation using the multiscale (*orange*) and mass action (*purple*) model calibrated without the tOPV data during a severe outbreak. Severe outbreaks were defined as having a transmission duration of at least 300 days. Susceptibility was defined as having a OPV-equivalent antibody titer < 8. The increase in susceptibility over time is due to waning immunity and births.

The first study goal was to generate synthetic populations using the household evolution model, which simulates household evolution following births, deaths, and marriage based on anthropological rules of household formation and secession (**Materials and Methods**). The household evolution model was calibrated to demographic health survey reports [22–24] that, on average, capture the household and community structure of rural Bangladesh. Household and community structure was defined and evaluated on four criterion: 1) the household size distribution (**Fig 2A**), 2) the age distribution of infants and their two youngest household contacts observed in the study (**Fig 2B**), 3) the population age pyramid (**Fig 2C**), and 4) the size distribution of the 45 villages assigned with routine bOPV immunization (**Fig 2D**). The contact age distributions (**Fig 2B**) were not used in fitting the model. Rather, the observed similarity between data and model derives from the specification of individual, age- and sex- specific fertility, mortality, and marriage rates.

Simulating the Bangladeshi age pyramid required additional information regarding historical fertility and mortality rates. Historical fertility and mortality rates were approximated by extrapolating the rates reported in the 2004, the year of the earliest obtainable report with both, and 2014 Bangladesh Demographic Health Surveys (BDHSS) [22,23]. This approximation is imperfect, as it underestimates the proportion of individuals under fifteen and overestimates the proportion over fifty. Nonetheless, the simulated age pyramid reproduced a similar flared-base at younger age groups, a defining characteristic of populations with rapidly declining fertility rates [23]. Future model fits could be improved by incorporating historical fertility and mortality rates from earlier timepoints.

To replicate the conditions of the Matlab study population prior to the mOPV2 campaign, we recapitulated the demographic and immune structure of the 45 villages assigned with bOPV routine immunization. The 22 villages assigned with routine tOPV immunization were excluded and residual tOPV-derived Sabin 2 transmission was not simulated. Infants were assigned OPV-equivalent antibody titers consistent with bOPV routine immunization and adults were assigned titers consistent with those expected of Matlab, Bangladesh (**S1 Text**).

### Calibrating vaccine virus transmission to the mOPV2 clinical trial

The next study goal was to test whether household and community structure affected transmission heterogeneity. We compared the model fits of the multiscale and mass action contact-based transmission models. These models depend on specifying daily contact rates of infected individuals. The multiscale model has four transmission rate parameters–one for each level of social hierarchy–and allows transmission to be defined by household and community structure. The mass action model assumes homogeneous transmission with a single transmission rate parameter. (**Materials and Methods, S1 Text**).

These models were calibrated to the eight cohort-specific shedding profiles collected after the mOPV2 campaign. Latin hypercube sampling and a pseudo-likelihood function to minimize the discrepancy between simulated and observed data were used to identify optimal parameters (**S1 Text**). These shedding profiles (**Table 1**) reflect vaccination with mOPV2 (cohorts one, four, and seven), transmission that mostly likely originated from a nearby shedding household or *bari* member (cohorts two, six, and five), and transmission that mostly likely originated from a village or non-village member (cohorts three and eight).

These shedding profiles alone were insufficient to reject homogeneous transmission as a possible transmission mechanism (**S1 Text**). Initial calibration attempts with the multiscale model were unsuccessful, as a wide variety of equally reasonable fits could be obtained with multiple combinations of household, *bari*, within-village, and inter-village contact rates. Under standard parsimony practices, this would suggest that household and community structure does not perturb transmission significantly enough to reject the assumption of homogenous transmission or mass action. Alternatively, it could suggest that these shedding profiles lacked the power to differentiate transmission between household, bari, village, or non-village members.

Fortunately, transmission following tOPV routine immunization during the enrollment period of the study provided an additional source of evidence to determine whether transmission is dependent on household community membership. From the small number of tOPV-derived transmission observed during the period prior to the mOPV2 campaign, we extrapolated bounds on the within- and between-village transmission rates for the mOPV2 campaign to serve as priors for model calibration (**S1 Text**). With these additional priors, the four-parameter multiscale model became identifiable (**S1 Text**). We tested three different transmission models: two mass action models, one with (**Fig 3**, *green*) and without the additional tOPV-derived transmission data (**Fig 3,** *purple*), and a multiscale model (**Fig 3**, *orange*) calibrated with the additional tOPV-derived transmission data.

When assessed on the cohort shedding data alone, the multiscale and mass action model calibrated without the tOPV-derived priors from the enrollment period provided reasonable model fits to cohort shedding data (**Fig 3**). The Akaike Information Criterion (AIC) for the multiscale model (**S1 Text**) was 2352.59 and 2355.40 for the mass action model calibrated without the tOPV data, and 2365.48 for the mass action model calibrated with the tOPV-derived priors. The better fit of the multiscale model was most prominent in cohort six, which monitored shedding in non-vaccinated household contacts of vaccinated infants and was thus most sensitive to household transmission. When assessed using all the evidence, the multiscale model was superior (AIC = 2350.17) to either of the mass action models, whose AICS were 2874.52 and 2380.87 with and without the tOPV-derived data. Taken together, these results show that poliovirus vaccine virus transmission in Matlab does depend on household community membership but that the cohort shedding data on its own lacked the power to show this.

The difference in model fit resulted from a mechanistic difference in how transmissions were distributed. Both mass action models were rejected due their inability to match the low levels of between- village transmission inferred from the tOPV-derived transmission data. The multiscale model predicted that 85% (83, 88) transmissions were made among *bari* and village members while both mass action models predicted >99% of events were made between non-village members. Mass action favors inter-village transmission because the chances of sampling a non-village member far exceeds the probability of sampling a household, *bari*, or village member (**S1 Table**).

### Forecasting vaccine virus transmission risk in Matlab, Bangladesh

We examined the consequences of the Switch on Sabin 2 vaccine virus transmission in Matlab. Three scenarios were examined: 1) Point importation: Sabin 2 vaccine virus transmission introduced by the accidental importation of a single Sabin 2 shedding infant, 2) Mass vaccination: Sabin 2 vaccine virus transmission following mOPV2 vaccinations campaign with up to 80% coverage in children under five years of age, and 3) cVDPV2 importation: cVDPV2 transmission following the accidental importation of a single cVDPV2 infected infant. To investigate the consequences of the Switch, these scenarios were performed in populations where routine Sabin 2 OPV vaccination had been discontinued for up to five years. Population-level immune profiles immediately before and up to forty years following the Switch are shown in **S2 Fig**.

### Quantifying point importation outbreak risk

Accurately quantifying and identifying populations with high outbreak probability is critical for preventing eventual poliovirus eradication and preventing cVDPV spread. We explored the practical consequences of inaccurately assuming homogeneous transmission when generating outbreak forecasts by generating two predictions: one using the fully calibrated multiscale model, and one using the mass action model calibrated without the tOPV-derived transmission data to determine whether the additional transmission heterogeneity imposed by household and community structure represented in the multiscale model was necessary for accurate risk assessment.

Both models predicted outbreaks with highly heterogeneous outcomes. As a whole, assuming mass action increased the probability of severe outbreaks with larger infection numbers and transmission durations (**Fig 4A–4C**). Under the multiscale model (**Fig 4B)**, point importations occurring five years after the Switch had a median transmission duration of 81 (9, 400) days of transmission, a maximum of 7 (1, 89) infections at the peak, and a total of 240 (8, 13000) infections.

When evaluating the outbreak risks associated with point importation, we reasoned that only the most severe outcomes would be detectable during routine surveillance and be informative to interventions. For the purposes of this study, a severe outcome was defined by an outbreak that persisted for at least 300 days. Under this definition, 0.03 and 0.177 the 2000 outbreaks simulated five years after the Switch under the multiscale model and mass action model were severe. Severe outbreaks under the multiscale model had an expected peak infection count of 98.0 (50.0, 128.0) and an expected transmission duration of 337 days (320.0, 361.0). The corresponding expected peak infection counts and transmission duration under the mass action model were 896.0 (789.0, 1030.0) infections and 498.0 (486.0, 511.0) days.

To determine whether the differences in model prediction reflected differences in transmission failure resulting from stochastic loss, high population-level immunity, or both, we examined the simulated immunity profiles during each point importation (**Fig 4D**). Under the multiscale model, the immunological profiles suggested that the vast majority of point importations failed due to stochastic transmission failure, as the outbreaks had no impact on population immunity and failed when 20% of the population was susceptible (OPV-equivalent antibody titer < 8).

Point importations under the mass action model were more complex. Outbreaks had no effect on population immunity during the first 100 days and point importations that failed during this period were likely the result of stochastic transmission failure. However, when conditioned on severe outbreaks, the simulations revealed a decrease in population immunity that was coincided with each simulation’s peak infection time. This immunological profile suggested that the outbreaks generated by the mass action were more strongly limited by the depletion of susceptible individuals in the population.

### Evaluating the benefits and risks associated with mass mOPV2 vaccination

**Fig 5:**
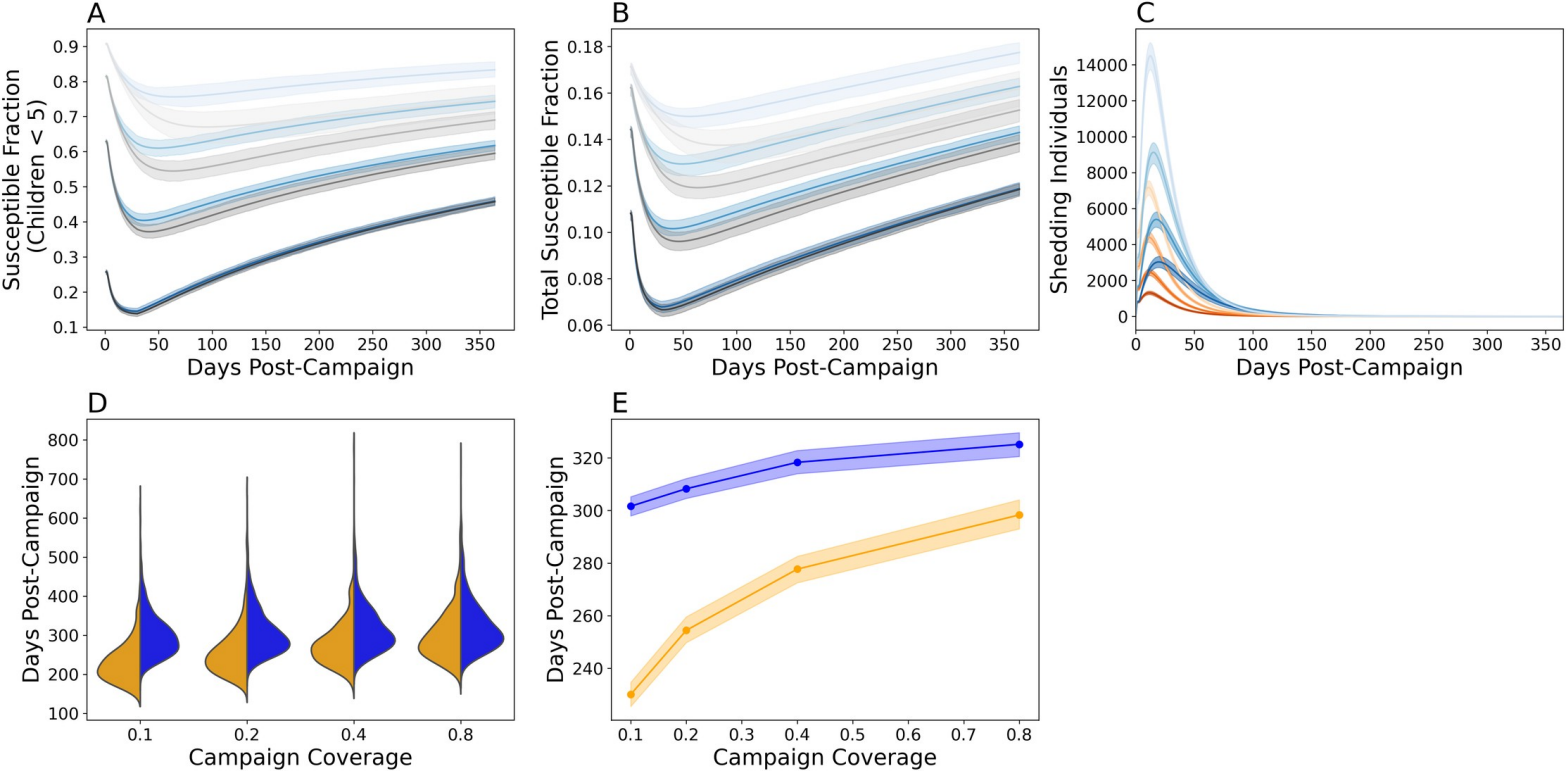
Simulating single-dose mOPV2 campaigns. The proportion of susceptible individuals with a serum neutralizing antibody titer under eight in **A)** children under five and **B)** the entire Matlab population during a single-dose mOPV2 campaign performed five years after the Switch. Simulation results using the multiscale model are shown in blue while those using the mass action model (calibrated without the tOPV) are shown in grey. **C**) Epi curves showing the number of infected individuals following the mOPV2 campaign before (*orange*) and five years after (*blue*) the Switch. For **A-C**), the darkness of the shading indicates the vaccination coverage in children under five. From darkest to lightest, the coverages were 80%, 40%, 20%, and 10%. 40% was the target coverage in the original mOPV2 campaign. Plotted are the mean expectations and the 95% prediction intervals obtained from 2000 simulation replicates. **D**) Violin plots showing the distribution of vaccine virus transmission duration before (*orange*) and five years after (*blue*) the Switch from 2000 simulation iterations. **E**) The expected shedding duration before (*orange*) and five years after (*blue*) the Switch. The solid line indicates the mean and the shading the bootstrapped 95% confidence interval of the mean.

To examine the benefits and risks of mass mOPV2 vaccination, we simulated mass vaccination campaigns with up to 80% coverage in children under five (**Fig 5**). These campaigns were evaluated for 1) their effectiveness at promoting population immunity (**Fig 5A and 5B**) and 2) the risks associated with reintroducing live poliovirus and vaccine-derived transmission (**Fig 5C–5E**). Interestingly, the disparity between the multiscale and mass action models decreased with vaccination coverage and was smallest when coverage was 80% (**Fig 5A and 5B,** *grey*). With the exception of the results shown in Fig 5A and 5B, all analyses were made using the multiscale model.

As expected, mass mOPV2 vaccination improved population immunity against type 2 poliovirus due to a mix of both direct vaccination and vaccine-derived virus transmission (**Fig 5A and 5B**). Vaccine virus transmission reduced the proportion of susceptible children under five by an additional 10–15% within the first month of the campaign. For an 80% vaccination campaign performed five years after the Switch, primary vaccination reduced the proportion of susceptible children under five to 0.257 (0.250, 0.263) with a further reduction to 0.145 (0.137, 0.154) after 29 days from vaccine virus transmission (**Fig 5A**). This proportion was similar to the proportion of susceptible children under prior to the Switch (**S2B Fig**). However, population susceptibility (**Fig 5A and 5B**) rebounded as vaccine virus transmission waned (**Fig 5C**) and the direct immunological benefits conferred by the vaccination campaign were reversed within the first year.

Two factors affected the duration of vaccine virus transmission: 1) the time since the Switch, and 2) the coverage level of the vaccination campaign. As expected, the expected vaccine virus transmission duration following each campaign has increased since the withdrawal of routine immunization (**Fig 5D and 5E**). Immediately after the Switch, increasing mOPV2 vaccination coverage from 10% to 80% caused the expected transmission duration to increase from 230.1 (225.68, 234.67) to 298.38 (293.29, 303.71). Five years after the Switch, the expected durations of vaccine virus transmission were 301.62 (297.76, 305.59) and 325.14 (320.99, 329.74) days for a 10% and 80% coverage campaign (**Fig 5E**).

**Fig 6:**
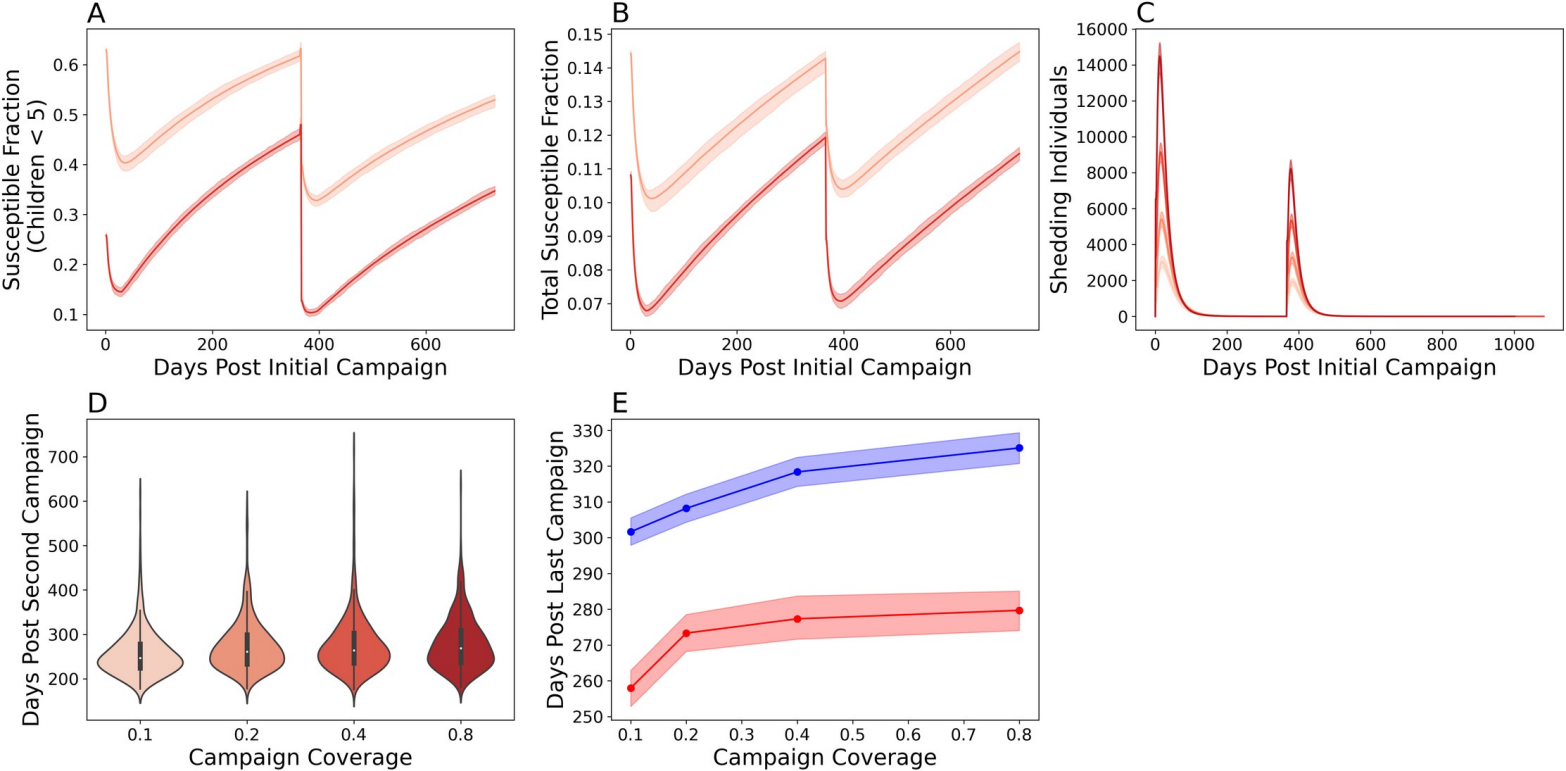
Simulating multiple, single-dose mOPV2 campaigns five years after the Switch. The proportion of susceptible individuals with a serum neutralizing antibody titer under eight in **A)** children under five and **B)** the entire Matlab population following a 40% (light red) and 80% (dark red) coverage campaign. **C**) Epi curves showing the number of infected individuals following the mOPV2 campaign following an 80% (darkest), 40%, 20%, and 10% (lightest) coverage campaign. **D**) Violin plots of the total shedding durations following the second campaign. The colors used in **C** are the same as that for **D**. **E**) The expected shedding duration after the first (blue) and second (red) mOPV2 campaign. Both campaigns had 80% coverage in children under five. The solid line indicates the mean and the shading the bootstrapped 95% confidence interval of the mean.

To examine the consequences of multiple, single-dose mOPV2 campaigns, we also examined a campaign with two single-dose mOPV2 interventions (**Fig 6**). The first occurred five years after the Switch and the second occurred one year after the first. Susceptibility among children under five accumulated less rapidly after the second campaign (**Fig 6A**). The proportion of children under 5 that were susceptible to infection was 0.48 (0.47, 0.49) one year after the first campaign and 0.33 (0.33, 0.35) one year after the second. The model also predicted less vaccine-virus transmission after the second campaign (**Fig 6C–6E**) in terms of the number of shedding individuals and the total duration of vaccine virus transmission. [17]

### Forecasting circulating vaccine-derived poliovirus transmission risk

Finally, we examined if the point importation of a cVDPV2 infected individual could cause an outbreak in Matlab. cVDPV2 infections were modeled by increasing the viral infectivity and shedding durations using the corresponding parameters for WPV described in [17]. Simulated cVDPV2 viruses were three times more infectious and caused infections that shed 1.4 times longer than Sabin 2 in immunologically naïve individuals [17].

**Fig 7:**
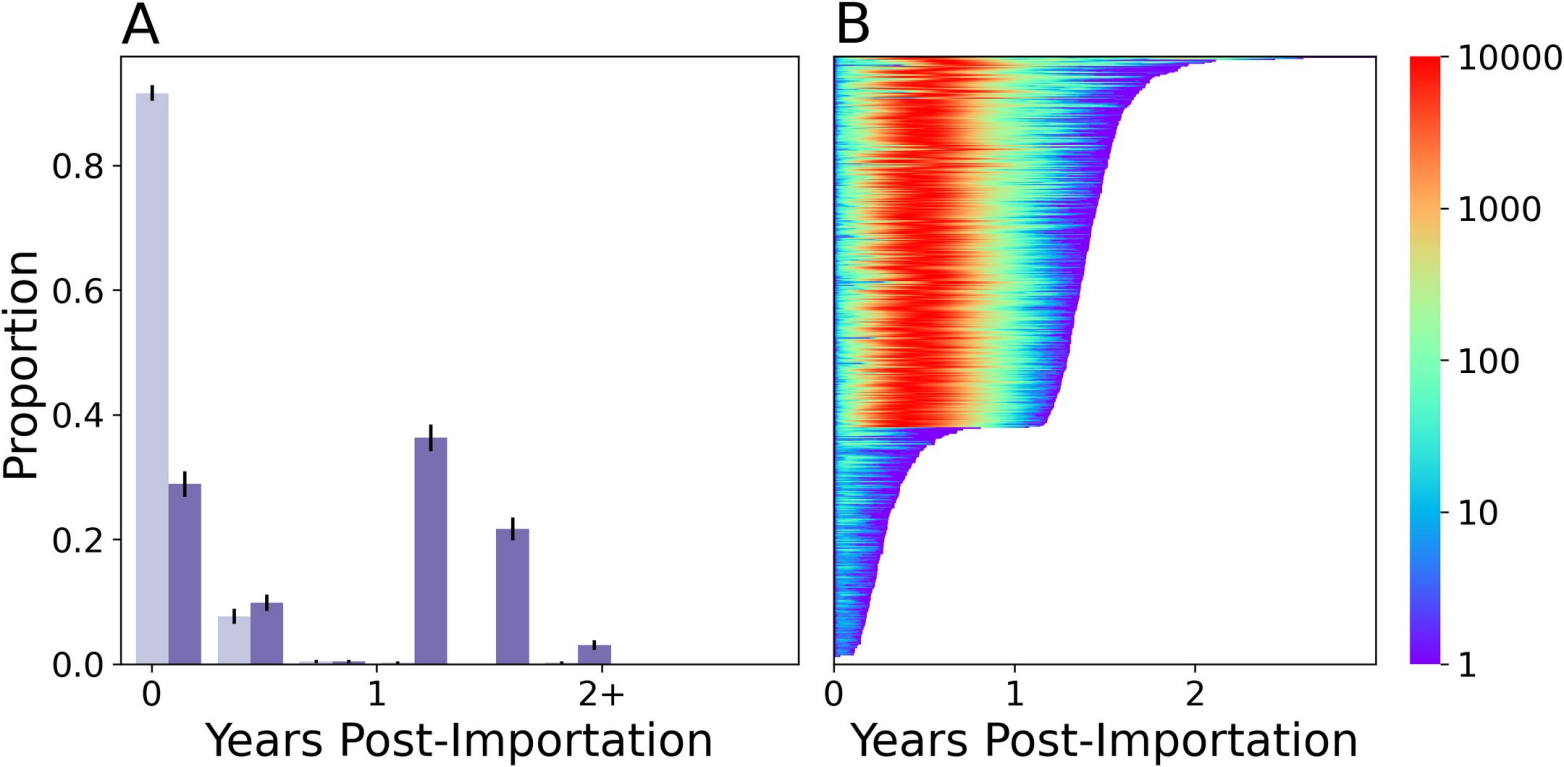
Extrapolating cVDPV2 vaccine virus transmission risk. **A**) Transmission persistence following a Sabin 2 point importation event before (year 0, *light purple*) and five years after the Switch (*dark purple*) from 2000 simulations. **B**) Lasagna plot of showing the total persistence times and number of infected individuals following a cVDPV2 point importation five years after the Switch using the multiscale model.

The model predicted that the cVDPV2 outbreak risk in Matlab has grown since the Switch. Before the Switch, the model predicted that most cVDVPV2 importations were self-limiting and ceased transmission within the first year (**Fig 7A**). 0.008 of the point importations resulted in severe outbreaks with a median peak infection count of 170 (72, 256). Five years after the Switch, the model predicted that 0.61 of point importations would result in severe outbreaks with a median peak infection count of 9534.0 (9447.0, 9668.0). While the expected transmission duration of these severe outbreaks was 516 (508, 524), 0.03 of the point importations sustained transmission for more than two years.

## Discussion

Rapid mOPV2-related risk assessments are needed to guide policy decision-making and ensure the continued safety and efficacy of OPV vaccines. To enable future risk assessment, we developed a new poliovirus transmission model that differs from previous dynamic poliovirus model [9,10,25,26] by integrating the biological aspects describing the immunity and shedding durations of infected individuals [17] with the social aspects of transmission described by household and community structure. Overall, the model showed that the decline in type 2 immunity following the Switch has elevated vaccine virus transmission and increased the frequency and severity of severe point importation outbreaks. The increased risk is particularly significant for cVDPV2 exportation, owing to the increase in infectiousness due to genetic reversion.[6,14]

For Matlab, we found that Sabin 2 transmission was congruent with a model centered on transmission between local *bari* and village members. Surprisingly, the multiscale model predicted that *bari* members have the highest burden of infection, which be partially due to the sharing of communal kitchens within each *bari*. This pattern of localized transmission is supported by cholera surveys in Matlab [27], which have reported strong spatial clustering of cases at distances less than two kilometers and within *baris* [28,29]. The cumulative effect of household and community structure is an increase in the stochasticity of transmission. Mass action underestimates stochastic transmission loss and risks over-estimating transmission when the initial infection count is small, such as during point importation or a low coverage vaccination campaign. Accurately characterizing the stochastic transmission heterogeneity is critical for risk assessments as populations with identical immunological histories will have different risks depending on how contact and or transmission structure influences stochastic transmission loss.

While our simulations showed that vaccine virus transmission during a mass mOPV2 campaign extends immunity beyond the primary vaccination recipients, this benefit is limited to the first few weeks after a campaign. The simulations predict that residual vaccine virus transmission can be maintained for months after the mOPV2 campaign. This transmission is too limited to impact population immunity but poses a significant risk for cVDPV2 emergence and exportation. These results are consistent with the tracing of cVDPV2 outbreaks to mOPV2 campaigns performed in other regions months after the original campaign ended [8]. Unsettlingly, the point importation simulations suggest that detected cVDPV2 outbreaks represent a small fraction of all importation events, and that vaccine virus exportation is common. Preventing the exportation of vaccine virus to non-intervention regions will be a major challenge for future poliovirus eradication campaigns.

The need to manage this risk supports the practice of using repeated, ideally high coverage mOPV2 campaigns that promote immunity and better limit vaccine-derived transmission by depleting the availability of susceptible individuals with low immunity, reducing the probability of new infection, and reducing the viral shedding concentration and durations of infected individuals [17] (**Supplemental**). A subject of future study would be to characterize the optimum frequency of vaccination campaigns that maximally utilizes the limited global stock of OPV while reversing the accumulation of susceptible individuals with as little residual vaccine-derived transmission as possible. However, these campaigns must be coupled with precautions designed to prevent vaccine-derived virus from escaping the intervention zone, as any OPV vaccine-based intervention strategy will always bear a significant risk of seeding other populations with vaccine virus as global type 2 immunity continues to decline. Heightened surveillance of regions proximal to the targeted intervention zone will be a necessary precaution and will need to detect any potential vaccine virus exportation early enough to prevent its spread.

Using the model to generate population-specific estimates of poliovirus transmission risk remains a challenge. A major goal of this study was to enable improved risk assessment in regions such as sub-Saharan Africa, Afghanistan, and Pakistan, where recurrent cVDPV2 outbreaks and sustained vaccine-derived transmission are prevalent and immunity to type 2 poliovirus has historically been low [30,31]. To our knowledge, it is unknown how heterogeneous transmission operates in these regions or whether certain regions are expected to have differing levels of transmission heterogeneity due to differences in household and community structure. While the clustering of households into *baris* made Matlab an ideal study location to examine the effects of household and community structure on poliovirus transmission, that same preference makes it difficult to assess whether poliovirus transmission in other regions is similarly impacted. Thus, assessing the impact of household and community structure and determining the extent to which mass action is inappropriate will be an important step for enabling accurate cVDPV2 outbreak and transmission risk in regions outside of Matlab.

In conclusion, our study provides a framework for generating population-specific assessments of OPV-related transmission risk and stresses the importance of household and community membership when modeling poliovirus transmission. We note that our current study underestimates the true risks associated with mOPV2 vaccination because the model does not yet simulate the vaccine virus attenuation reversal due to genetic reversion [5]. Integrating vaccine virus evolution will be necessary to truly assess the risks associated with cVDPV2 emergence and the duration of vaccine-derived virus circulation following mOPV2 usage. Future iterations of the model could also explore [5] the use of the novel oral polio vaccines [32], and evaluate whether the incorporation of other social factors, such as preferential contact structures based on age, occuputation, and time, would improve model fit [33]. Other applications include replacing the poliovirus infection to simulate the transmission of other fecal-oral diseases, such as typhoid, shigellosis, and other diarrheal diseases [34–36].

## Supporting information

S1 Text Supporting Information. Supporting information containing details regarding model design and calibration.
(DOCX)

S1 Data Parameter Table. List of the parameters used in the model.
(XLSX)

S1 Fig mOPV2 clinical trial design. A) Routine immunization and enrollment phase and B) the mOPV2 campaign and the 22 week longitudinal surveillance period. The tOPV villages was excluded from simulation due to the larger potential of unmodeled vaccine transmission from routine immunization.
(TIF)

S2 Fig Population Immunity Following Vaccination Cessation. **A**) Average immunity (log2 OPV-equivalent antibody titers) against Sabin 2 in our populations immediately after (zero years), one, five, ten, and 40 years after the Switch. Solid line indicates the population average and the shading the boundaries of the middle 95^th^ percentile. Note the age-structured erosion of population immunity due to new births and immune waning. **B**) The proportion of susceptible children under five (*blue*) and the proportion of susceptible individuals in the population (*orange*) against years since the Switch.
(TIFF)

S3 Fig Fertility and mortality fits in the demographic model. **A-D**) The birth interval periods for individuals aged **A**) 15–19, **B**) 20–29, **C**) 30–39, and **D**) 40–49 in 2014. The number of children per married female in 2014 (**E-H**) and 2004 (**I-L**) for individuals aged **E/I**) 15–19, **F/J**) 20–24, **G/K**) 25–29, and **H/L**) 30–34. The age specific fertility rate (ASFR, number of births per 1000 individuals) in 2014 (**M**) and 2004 (**N**). For subplots **A-N**, simulated results are presented in orange, data from 2014 are presented in blue, and data from 2004 are presented in teal. Error bars in all bar plots indicate one binomial standard error from the mean. The shading in plots **M** and **N** indicate the middle 95% confidence intervals of the data. Simulated confidence intervals were generated by replicating the simulation 100 times. **O-N**) The sex-(blue = male, pink = female) and age-specific mortality rates in 2014 (**O**) and 2004 (**P**). WHO mortality rates are represented by dots.
(TIF)

S4 Fig Immune boosting. **A**) Ordinary least squares fit relating pre-exposure log2 antibody titers to the ratio of post-exposure to pre-exposure antibody titer observed in 1953 Louisiana.[37] Overlapping points are randomly jittered to better represent point density. Line represents the ordinary least squares fitted equation. **B**) Residual plot of our ordinary least squares fit. The fan-shaped distribution is classic signature of heteroskedasticity. **C**) Fitted ordinary least square function relating variance with pre-exposure titer. **D**) Final immune boosting model predictions (*orange*) compared against the original data (*blue*).
(TIF)

S5 Fig Multiscale Parameter Search and Identifiability. Profile likelihoods of for the four mass action transmission parameters: **A)** *β_hh_*, **B/E**) *β_bari_*, **C/F**) *β_village_*, and **D/G**) *β_intervillage_*. **A-D** were generated without the priors for within- and between- village transmission from the tOPV data. Note the multiple peaks present in **D** and the non-monotonic decrease in negative log-likelihood in **B**. **E-G** were generated with the priors. For **E-G**, the black profile likelihood represents the profile used to parameterize the multiscale model. The dark and light red likelihoods show the profile likelihoods assuming a higher and lower confidence in the accuracy of the tOPV-derived priors.
(TIF)

S6 Fig Mass Action Parameter Search and Identifiability. Profile likelihoods for the single-parameter mass action model (*β_ma_*) without (**A**) and with (**B**) the priors for within- and between- village transmission from the tOPV data.
(TIF)

S1 Table Contact probabilities. The average number of household, bari, village, and non-village members reletive to each infected individual. The probability of contacting each of these individuals under mass action was quantified as the average number of individuals in each category divided by the average number of individuals in the population. The expected number of contacts (E(Contact)) was quantified by taking these probabilities and multiplying it with the number of contacts an individual infection makes using the β_ma_ parameter of the mass action model calibrated without the tOPV data. The expected number of contacts for the multiscale model were the same as the *β* parameters identified for that model.
(XLSX)
