# Supplementary material for: Multiscale model for forecasting Sabin 2 vaccine virus household and community transmission
Source: PLoS Comput Biol. 2021 Dec 21;17(12):e1009690. doi: 10.1371/journal.pcbi.1009690 (PMC8726461; doi:10.1371/journal.pcbi.1009690)
Supplement: S1 Text — Supporting information containing details regarding model design and calibration. (DOCX) [file pcbi.1009690.s001.docx]

Supporting Information

**Modeling Individuals**

**Individuals**

Agents in our model represent individuals defined by their age, sex, marital status, and immunity (see below, ***Supplemental* – Modeling** **Immunity**). Female individuals are also defined by their fertility, which determines the probability of birth given marital status and age. Death occurs when individuals reach a pre-determined death age based on calibrated mortality rates. A visual summary of model calibration fits with regards to fertility and mortality are presented in **S3 Fig.**

**Fertility**

Fertility is determined using a joint probability function conditioned on four factors:

|  | $P\left( Birth \right)=P\left( Birth \vert age,n_{children},t_{birth}, marriage \right)$ | (1) |
| --- | --- | --- |
|  | $P\left( Birth \vert x=age \right)= \left\{ \begin{aligned} Ax^{4}+Bx^{3}+Cx^{2}+Dx+E if 50<x<15 \\ 0 else \end{aligned} \right.$ | (2) |
|  |  |  |

|  | $P\left( Birth \vert x=n_{children} \right)= \left\{ \begin{aligned} \frac{c}{1+e^{-k\left( x-x_{0} \right)}}+y_{0} if x>1 \\ 1 else \end{aligned} \right.$ | (3) |
| --- | --- | --- |
|  |  |  |
|  | $P\left( Birth \vert x=t_{birth}, age \right)= \left\{ \begin{aligned} \alpha\left( \frac{k_{age}}{\lambda_{age}} \right)\left( \frac{x}{\lambda_{age}} \right)^{k_{age}-1}\left( 1-e^{(-{x/{\lambda_{age})}}^{k_{age}}} \right)^{\alpha_{age}-1}e^{(-{x/{\lambda_{age})}}^{k_{age}}} if x > 1 \\ 0 else \end{aligned} \right.$ | (4) |
|  |  |  |
|  | $P\left( Birth \vert marriage \right)= \left\{ \begin{aligned} 1 if married \\ 0 else \end{aligned} \right.$ | (5) |

Equation 2 is a polynomial equation that represents the maximum, base fertility of a married female for her age. The number of previous births (n_children_) and birth interval period (t_birth_) are modifiers that decrease fertility based on the individual’s previous history. P(Birth|n_children_) is a sigmoid function that penalizes having additional children and P(birth| t_birth_) is the probability density function of an exponentiated-weibull distribution that prevents births from occurring too close to one another. The parameters of this exponentiated-weibull distribution are age-specific to incorporate the increasingly large birth intervals associated with age. We assume that only married females give birth. P(Birth|marriage) is a binary flag that prevents single or widowed females from giving birth. Parameter estimates are in the **S1 Data.**

**Birth Interval Calibration**

The birth interval function describes the expected wait-time between births and is defined by an exponentiated Weibull distribution. We calibrated this function to the data reported in the 2014 Bangladesh Demographic Health Survey. Reported birth intervals were categorized into six bins (7-17 months, 18-23 months, 24-35 months, 36-47 months, 48-59 months, and 60+ months) for four age groups (15-19, 20-29, 30-39, and 40-49 years of age). We independently fit an exponentiated Weibull function to each of the age groups to obtain four sets of age-group specific parameters by maximizing a multinomial likelihood function:

|  | $L\left( \lambda_{age},k_{age},\alpha_{age} \right)=\prod_{i=1}^{6} p_{i, age}^{x_{i, age}}$ | (6) |
| --- | --- | --- |
|  | $p_{i,age}=\int_{t_{1}}^{t_{2}} P\left( Birth \vert x=t_{birth},t \right)dt$ | (7) |

where p_­i_­ is the proportion of births occurring in the time interval [t1, t2] in months for that age group. Parameter estimates are in the **S1 Data.**

**Marriage Calibration**

To incorporate marriage into our fertility calibrations, we defined marriage as:

|  | $P\left( marriage \vert x=age \right)=\int_{t-1}^{t} \frac{kp_{0}e^{rx}}{k+p_{0}e^{rx}-1}$ | (8) |
| --- | --- | --- |

where t is the current timestep and the other parameters are associated with a logistic growth curve (Verhulst equation). We calibrated our marriage function to the proportion of married individuals by age data in the 2014 Bangladesh Demographic Health Report using the a non-linear least-squares optimizer (scipy.optimize.curve_fit). Note that this equation is only used for calibration. The actual marriage rates in our full model are influenced by household and population dynamics (***Supplemental - Household demographic structure****).* Parameter estimates are in the **S1 Data.**

**Base Fertility and Child Preference Calibration**

For the remaining parameters, we calibrated our fertility function to the lifetime number of children per married female and the age-specific fertility rates reported in both the 2004 and 2014 Bangladesh Demographic Health Surveys. Reported lifetime children numbers per married female were split into eight different age groups (15-19, 20-24, 25-29, 30-34, 35-39, 40-44, 45-49 years of age). We calibrated our model against age groups less than 35 years of age because older age groups are more likely to be influenced by past fertility trends.

By assuming independence between each of the components of our fertility equation, our birth function (equation 1) can be rewritten as:

|  | $P\left( Birth \right)=P\left( Birth \vert age)*P\left( Birth \vert n_{children} \right)*P(Birth\vert t_{birth} \right)*P\left( marriage \vert age \right)$ | (9) |
| --- | --- | --- |

The parameters for $P(Birth|t_{birth})$, the birth interval equation (equation 4) and $P\left( marriage | age \right)$, the marriage probability function (equation 8),were independently calibrated.

For the remaining parameters associated with the base fertility rate (equation 2) and the child preference function (equation 3), we created a simplified, agent-based fertility simulator consisting of 10,000 female individuals. Simulated individuals track the number of previous births, the time since most recent birth (birth interval), age, and marital status. Individual starts at age = 15 (the minimum fertility age) and are aged using one-year timesteps until they reach age 50, which we assumed to be the maximum fertility age. At each time step, we used our modified birth function (equation 9) to determine whether an individual will give birth given their age and past birth history. We then compared our simulated estimates to the reported data using a population Monte Carlo (PMC) Approximate Bayesian Computation (ABC) to obtain posterior parameter estimates. We did not re-calibrate our birth interval parameters. Our PMC contained 3000 particles and was iterated four times. Initial priors were drawn from normal distributions whose parameters were loosely based off hand calibration attempts. After each iteration, we defined distance as the Kullback-Leibler divergence and modified our acceptance threshold such that only the top 30, ten, and five percent of the distance distribution would be accepted for the second, third, and fourth iteration. The median posterior value for each parameter were used as our calibrated point estimates in our full model. Parameter estimates are in the **S1 Data.**

**Defining Mortality**

Individuals in our model are assigned a death age at birth. Mortality rates were defined using a piecewise function:

|  | $P\left( death \vert sex, x=age \right)= \left\{ \begin{aligned} max\left( m,e^{\left( B_{child}+A_{child}x \right)} \right) if x<12 \\ max\left( m,e^{\left( B_{elder}+A_{elder}x \right)} \right) if x>30 \\ m else \end{aligned} \right.$ | (10) |
| --- | --- | --- |

where m is the minimum mortality rate and calibrated to both the 2004 and 2015 WHO life tables for Bangladesh. These life tables reported the mortality rate per 10,000 individuals, split into different age groups. Individual fits were made for male and females. Linear regression models were fit to the natural log mortality rate using a non-linear least squares optimizer (scipy.optimize.curve_fit). We assume a minimum mortality rate of 0.001 (the minimum number of possible deaths per 1000 individuals) to ensure that death is possible at all ages. Point estimates for the linear regression model are in the **S1 Data.**

**Historical Fertility and Mortality Projection**

To approximate the rapid, historical decline in fertility and mortality, we instituted a two-phase burn-in lasting 180 years. This burn-in was divided into two phases: t_historical_, a 140-year historical period with high fertility and mortality rates, and t_transition_, a 40-year transition period where fertility and mortality rates decline to their 2014 levels. We defined the historical fertility rate as:

|  | $P\left( Birth_{historical}\vert age,n_{children},t_{birth}, marriage,t \right)=H_{fertility}(t)* P\left( Birth_{2014} \vert age,n_{children},t_{birth}, marriage \right)$ | (11) |
| --- | --- | --- |
|  | $H_{fertility}\left( t \right)=\left\{ \begin{aligned} \frac{t}{40}\left( 1-\omega_{fertility} \right)+ \omega_{fertility} if t<40 \\ \omega_{C} else \end{aligned} \right.$ | (12) |
|  | $\omega_{fertility}\left( x=age \right)= \omega_{C}\left( \omega_{a}x + \omega_{\beta} \right)$ | (13) |

Historical fertility rates are obtained by adding an age and time-dependent multiplier, H, to the 2014 fertility rate. t is the number of years prior to 2014. Assuming only the base fertility rate (equation 2) changes through time, $\omega$ represents the per-decade increase in base fertility for each decade prior to 2014. $\omega_{C}$ is the maximal historical increase in fertility, which was set to three to make fertility rates increase from 2 to 6 (an approximation of the historically high fertility rates in Bangladesh) children per female. $\omega_{a}$ and $\omega_{\beta}$ are parameters to a standard linear function and were calibrated to the 2014:2004 base fertility ratios in the 2014 and 2004 Bangladesh Demographic Health Survey data using a non-linear least squares optimizer (scipy.curve_fit). Point estimates for $\omega_{C}$ = 3,$\omega_{a}=0.85,$ $\omega_{\beta}$ = 0.18. The 2004 Bangladesh Demographic Health Survey was the earliest, obtainable survey at the time of this study.

Similar logic was applied to the mortality rates, except that we scaled it proportionally to the 2004 and 2014 mortality rates. This did not have a significant impact, as the mortality rates between these two time periods were relatively the same. Here, mortality was capped at their 2004 levels.

| $P\left( death_{2014}\vert age, sex, t \right)=H_{death}(t)*P\left( death_{2014}\vert age, sex \right)$ | (14) |
| --- | --- |
| $H_{death}\left( t \right)=\left\{ \begin{aligned} \frac{t}{40}\left( P\left( death_{2014}\vert age, sex \right)-P\left( death_{2004}\vert age, sex \right) \right)+ P\left( death_{2004}\vert age, sex \right) if t<40 \\ \alpha_{C} else \end{aligned} \right.$ | (15) |
| $\alpha_{C}= P\left( death_{2004}\vert age, sex \right)$ | (16) |

These equations were calibrated to the WHO Bangladesh life tables for 2014 and 2004 using the same procedure as above. Note that this extrapolation does not account for changes in fertility and mortality outside of the ten-year window between 2004 and 2014. Major events such as the Bangladesh famine of 1974 were not taken into account.

**Supplemental – Household evolution model**

Traditional, rural Bangladesh households are patriarchal, stem families. Stem families are a type of family system in which one child (commonly the firstborn son) stays within the family home while other children move out to live in with their in-laws or to start households of their own. Households in our model are represented as trees and based off an anthropological framework described in [1]. Each node represents either a single, unmarried individual or a marital unit (one male and female). Although other types of marital units exist in traditional Bangladeshi society (particularly polygynous unions), they were not simulated. The root node of each tree represents the founding couple. The male in the root node represents the patriarch. Newly born individuals are assigned a new node connected to its parental node. This allows households to grow organically while preserving the hierarchical relationship between household members.

Nodes can have one of six statuses: single (never married), married, widow, widower, orphan, and dead. Dead terminal nodes are pruned at each timestep while dead internal nodes are preserved to maintain downstream hierarchies. We define orphans as individuals whose immediate parents have died and who are younger than the minimum eligible marriage age. Widowers, orphans, and single females are considered dependent states and preferentially kept in pre-existing households (see below—*Succession and household splitting*). Although this framework was designed with Bangladesh society in mind, it can be reasonably extended to other traditional, rural societies where large extended families are common. However, because our model equates household with families of related individuals, it does not simulate living arrangements resulting from non-familial roommate situations (ie working camps or dormitories).

Our model updates individuals, households, *baris*, and villages using discrete time steps. During each timestep, our model:

1. Accounts for births and deaths
2. Updates the age, fertility, and immunity of living individuals
3. Updates households by removing dead individuals
4. Updates *baris* by removing dead households and creating new households generated during move out or succession events
5. Updates villages by removing dead *baris* and creating married couples by moving eligible, single females to their spouse’s household. Newly married partners are sourced from different *baris*.

**Marriage**

Newly born individuals are assigned an earliest marriage age drawn from normal distributions whose mean and standard deviations were obtained from the 2014 Bangladesh Demographic Health survey. The average earliest marriage ages are 27.3 and 19.3 for males and females respectively. Once eligible, individuals are randomly paired with a partner of the opposite sex. Eligible partners are drawn from the village but excludes *bari* members to avoid incest. During marriage, females are removed from their original household and added to their spouse’s node. Only females in married nodes can give birth. Should one member of the marital unit die, the node status is updated to either widow or widower. We do not allow remarriage in our model.

**Succession and household splitting**

The patriarchal, stem structure of Bangladesh households influences household composition through time, particularly with regards to inheritance and succession [2]. Traditional Bangladesh households are governed by a patriarch (usually the eldest male) from which all other individuals either descend from or added through marriage. Inheritance and succession favor males, and elders preferentially live with their eldest son. This hierarchy reorganizes itself upon patriarch death, with the eldest son assuming the role of patriarch; younger male siblings leave after marriage to form their own households in the *bari*.

Succession is the primary mechanism for household formation in our model. Our household model initiates succession when the patriarch in the root node dies. During succession, our model:

1. Identifies viable subtrees within the original household tree. Viable subtrees are identified by traversing the household tree in level order (visiting every node on a level before going to a lower level, excluding the root node) and identifying the inner-most, still-living nodes. Internal nodes identified through this process represent married couples with descendants or widows/widowers with descendants. Terminal nodes represent childless couples, unmarried individuals of eligible marriage age, childless widows/widowers, or orphans whose parents have died but are too young to marry.
   1. If the root node still contains a living member (the matriarch), it is treated as a terminal node containing a childless widow.
2. Creates new households by extracting viable subtrees from the original household. Subtrees are rooted on the inner-most, still-living nodes identified in step one. Those rooted on couples (with or without children). Those rooted on single individuals, widows or orphans are considered unviable (see step three). Once extracted, these new trees represent new households within the bari. One of these trees is randomly chosen to be the successor and inherit the original household.
3. Reassigns non-viable subtrees to the newly created successor household as immediate descendants of the root.
   1. If no viable successor household was created, non-viable subtrees are assigned to a random pre-existing household in the bari. Only if no pre-existing households exist will these non-viable subtrees be used to create new households.

Our succession framework preserves the familial relationships between individuals within subtrees but does not preserve the familial relationships between subtrees (cousin, uncle, and other distant relationships are more likely to be lost). Despite this, we can still identify whether individuals are generally related to one another as new households are placed in the original *bari*. Our framework also prevents elders, widowers, orphans from living on their own. This is motivated by the living arrangements of these individuals in traditional, rural Bangladesh society. Bangladeshi elders almost exclusively live with their descendants, with a strong preference for the eldest son [2]. Widowers have historically moved back to their childhood household. Although our model does not simulate these dynamics exactly, allowing these individuals to live on their own resulted in a higher proportion of single households than expected for Bangladesh society. Only after incorporating step three were we able to reduce the proportion of simulated single households to match that of Bangladesh society. In many ways, this mirrors the difficulty of real societies to care for marginalized or otherwise non-independent members.

**Supplemental: Modeling** **Immunity**

We model immunity using the mathematical model described in [3]. Our model relates oral susceptibility to infection, shedding duration, and viral shed concentrations to pre-exposure immunity ($N_{ab_{pre}})$; high $N_{ab_{pre}}$reduces oral susceptibility to infection, shedding duration, and viral shed concentrations. Our model also allows for waning immunity, which depends on the peak post-infection immunity ${(N}_{ab_{peak}})$. Immunity is defined as the OPV-equivalent antibody titer, an indirect measure of immunity representing the serum neutralizing antibody titers due to OPV immunization or natural wild poliovirus infection. OPV induced antibody responses are predictive of fecal shedding and susceptibility while inactivated poliovirus (IPV) induced responses are not [4–8]. Equations for shedding duration after OPV challenge, poliovirus stool concentrations, oral susceptibility to infection, and waning immunity were taken from supplemental equations S1-S6 from the previous study [3] and are copied here for clarity. Parameter estimates are available in S1 Dataset.

**Oral susceptibility**

Oral susceptibility is modeled as a dose-response relationship between infection, oral poliovirus ingestion, and pre-exposure immunity.:

|  | $P\left( infection \vert dose,N_{ab_{pre}}, strain \right)=1-\left( 1+\frac{dose}{\beta_{strain}} \right)^{-\alpha\left( N_{ab_{pre}} \right)^{-\gamma}}$ | (11) |
| --- | --- | --- |

where dose refers to the viral dose and $N_{ab_{pre}}$ refers to pre-exposure immunity. $\alpha$ and $\beta_{\mathrm{strain}}$ are standard beta-Poisson parameters and $\gamma$ captures the reduction in infection probability with increasing immunity. $\beta_{\mathrm{strain}}$ is type-specific and different for Sabin 1, Sabin 2, Sabin 3, and wild poliovirus. Parameter estimates for Sabin 2 and WPV are found in the supplemental of the original paper [3] and in S1 Dataset.

**Shedding Duration and Shedding concentration**

We assumed a log-normal survival distribution for shedding duration:

|  | $P\left( shedding at t \vert N_{ab_{pre}};infected at t=0 \right)=\frac{1}{2}\left( 1-erf\left( \frac{ln(t) - (ln(\mu) - ln(\delta)log\_2 (N_{ab_{pre}})}{\sqrt{2}ln\left( \sigma\right)} \right) \right)$ | (12) |
| --- | --- | --- |

where $\mu$ is the median duration in days for immunologically naiive individuals ($N_{ab}=1)$, $\delta$ describes the decrease in median duration with increasing immunity, and $\sigma$ describes the shape of the distribution. Infection durations (t_duration_) are assigned at the start of the infection and determined by sampling from the inverse distribution. Once infection age exceeds infection duration, individual cease to shed virus and are considered uninfected.

To model viral load over time, we assume a quasi-log-normal shedding profile:

|  | $concentration(t \vert N_{ab_{pre}}, age) = max({10}^{2.6},$  $(peak CID50/g\vert N_{ab_{pre}}, age) * \left( \frac{exp(\eta- \frac{v^{2}}{2}-\frac{\left( ln\left( t \right)- \right)^{2}}{2\left( v+\xi ln\left( t \right) \right)^{2}}}{t} \right))$ | (13) |
| --- | --- | --- |
|  |  |  |
|  | $log_{10}\left( peak CID50/g \vert N_{ab_{pre}},age \right)=\left( 1-klog_{2}\left( N_{ab} \right) \right)log_{10}\left( peak CID50/g \vert N_{ab_{pre}}=1,age \right)$ | (13a) |
|  |  |  |
|  | $log_{10}\left( peak CID50/g \vert N_{ab_{pre}}=1,age \right)= \left\{ \begin{aligned} S_{max} \\ \left( S_{max}-S_{min} \right)exp\left( \frac{7-age}{\tau} \right)+S_{min} \end{aligned} \right.$ | (13b) |
|  |  |  |

Shedding concentrations are evaluated at time points, t, falling within the interval (0, t_duration_]. Parameter estimates and further details are found in the supplemental of the original paper [3] and in S1 Dataset.

**Immune Waning**

Immune waning is modeled as a power law:

|  | $N_{ab}\left( t \right)=max\left( 1,N_{ab_{\mathrm{peak}}}t^{-\lambda} \right)$ | (14) |
| --- | --- | --- |

where t is measured in months and $N_{ab_{\mathrm{peak}}}$ is the peak post-infection immunity. Parameter estimates are found in the supplemental of the original paper [3] and in S1 Dataset.

*Immune boosting*

Previously, we inferred $N_{ab_{\mathrm{peak}}}$ based on the shedding durations of individuals whose vaccination history was known. Conditioning our analysis to individuals with known vaccination histories allowed us to infer $N_{ab_{\mathrm{peak}}}$from individuals with multiple reinfection histories (*e.g.* a 3x bOPV vaccination course) without having to specify the relationship between $N_{ab_{\mathrm{pre}}}$ and $N_{ab_{\mathrm{peak}}}$. However, this approach was untenable for this study because individuals in Matlab, Bangladesh have complicated immune histories due to overlapping vaccination campaigns and because we wanted to dynamically model immune dynamics following transmission and potential reinfection. To dynamically model reinfection, we needed to quantify the boost in immunity (θ) following infection. Previous serology and viral shed studies strongly suggest θ diminishes with higher pre-exposure immunity [9,10].

We defined $N_{ab_{\mathrm{peak}}}$ and θ as:

|  | $N_{ab_{\mathrm{peak}}}=N_{ab}* \theta\left( N_{ab} \right)$ | (15a) |
| --- | --- | --- |
|  |  |  |
|  | $log(\theta\left( N_{ab} \right))= a+blog_{2}\left( N_{ab} \right)$ | (15b) |

where N_ab_ represents the pre-exposure antibody titer and θ is the boost response measured in log2 units. We calibrated θ to the post-exposure antibody ratios obtained from sera collected from 150 newborn infants monitored for poliovirus infection in 1953 [10]. Post-exposure antibody ratios are defined as the ratio between post-exposure and pre-exposure antibody titers and a direct measurement of immune boost. We first fit an ordinary least squares model to the post-exposure antibody ratios against the pre-exposure antibody titers, which revealed a negative correlation between θ and log2 $N_{ab}$ but with heteroskedastic variance (**S4 Fig**).

The heteroskedasticity associated with high $N_{ab}$ could be due to biological factors, such as immune exhaustion, or an artifact due to limit of quantification issues associated with sampling methodology. To differentiate these two, we split θ into two components, θ_bio_ and θ_sampling_ where:

|  | $\bar{\theta}\left( N_{ab} \right)= \bar{\theta}_{bio}\left( N_{ab} \right)+\bar{\theta}_{sampling}$ | (16) |
| --- | --- | --- |
|  | $\bar{\theta}\left( N_{ab} \right)= \alpha+\beta log_{2}\left( N_{ab} \right) +\bar{\theta}_{sampling}$ | (16a) |
|  | $Var(\theta\left( N_{ab} \right))=\gamma+ \delta log_{2}(N_{ab}) + Var(\theta_{sampling})$ | (17) |

For θ_bio_, we assumed that both mean and variance decreased linearly with N­_ab_ but that the mean and variance of θ_sampling_ were constant. We evaluated the six parameters in equations 12-13 (α, β, γ, δ, $\bar{\theta}_{sampling}$, $Var(\theta_{sampling})$) using a joint log-likelihood function. Our log-likelihood function is evaluated by splitting the serum antibody responses into two categories: seroconverted responses and non-seroconverted responses. We defined seroconverted individuals as those with post-exposure to pre-exposure titer ratios of at least four.

|  | $logL=logL_{\mathrm{seroconverted}}\left( \alpha,\beta,\gamma,\delta,\bar{\theta}_{sampling},Var\left( \theta_{sampling} \right) \right)+logL_{\mathrm{nonseroconverted}}\left( \bar{\theta}_{sampling},Var\left( \theta_{sampling} \right) \right)$ | (18) |
| --- | --- | --- |
|  |  |  |
|  | $logL_{s=seroconverted}= \sum_{N_{ab}}^{k_{s}} \left[ -\frac{n_{j_{s}}}{2}(log(2\pi) + log(Var(\theta\left( N_{ab} \right))) - \sum_{i}^{n_{N_{ab}}} \frac{1}{2{Var(\theta\left( N_{ab} \right))}^{2}}\left( x_{i}-\bar{\theta}\left( N_{ab} \right) \right)^{2} \right]$ | (18a) |
|  | $logL_{n=nonseroconverted}= \frac{n_{non}}{2}(log(2\pi) + log(Var\left( \theta_{sampling} \right)) - \sum_{i}^{n_{non}} \frac{1}{2Var\left( \theta_{sampling} \right)}\left( x_{i}-\bar{\theta}_{sampling} \right)^{2}$ | (18b) |

where k_s_ represents the binned N_ab_ categories reported in the data, n_Nab_ is the number of data points in in the N_ab_ bin, and n_non_ is the total number of datapoints in the non-seroconverted dataset. Our log-likelihood is the sum of two gaussian log-likelihoods, one for the seroconverted data and one for the non-seroconverted data. Our log-likelihood function assumes that changes in nonseroconverted individuals are due to sampling methodology while changes in seroconverted individuals is due to a combination of both sampling methodology and real biology.

Once mle estimates for $\bar{\theta}\left( N_{ab} \right)$ and $Var(\theta\left( N_{ab} \right))$ were obtained, we defined peak post-exposure immunity as:

|  | $N_{ab_{peak}}=N_{ab}e^{T}$ | (19) |
| --- | --- | --- |

where T is a random value drawn from a normal distribution with mean $\bar{\theta}\left( N_{ab} \right)$ and variance $Var\left( \theta\left( N_{ab} \right) \right)$. Parameter estimates are in the **S1 Data.**

**Initializing population-level immunity**

**Infants**

We assumed that pre-mOPV2 challenge immunity in infants was defined by a bOPV vaccination regiment with either 1x or 2x IPV given at ages six, ten, and 14 weeks of age. Despite not containing live Sabin 2 poliovirus, bOPV does induce a small amount of heterotypic immunity against Sabin 2. The amount of heterotypic immunity can be inferred from the shedding duration of infants challenged with mOPV2. To simulate this, we devised a reinfection model where infants are administered a vaccine-equivalent dose of Sabin 1 poliovirus (10^6^ infectious viruses) at six, ten, and 14 weeks of age. We assumed bOPV-induced immunity could be simulated as monotypic Sabin-1 immunity with a reduced probability of infection. At 18 weeks of age, infants were then challenged with mOPV2. We assumed infants were immunologically naiive prior to six weeks of age and that Sabin 1 shedding durations, shedding concentrations, immune boosting, and immune waning were identical to those of Sabin 2 [3]. To simulate lower heterotypic immunity, we modified the oral susceptibility equation (equation 11) by introducing multiplicative modifier, $\rho$, that reduces infection probability:

|  | $P\left( infection \right)=P\left( infection \vert dose,S1,N_{ab} \right) \rho$ | (20) |
| --- | --- | --- |

We estimated $\rho$ using our reinfection model by calibrating it to the Sabin 2 shedding duration of infants challenged with mOPV2 using a PMC-ABC with 1000 particles and four iterations. Distance was defined as the squared difference between simulated and empirical shedding prevalence collected weekly for five weeks post-mOPV2 challenge. Each iteration modified its acceptance threshold such that only the top 30, ten, and five percent of the distance distribution were accepted for the second, third, and fourth iteration.

**Non-Infants**

We assumed that pre-mOPV2 challenge in non-infants resulted from a complex immune history due to repeated vaccination or secondary transmission exposure from multiple vaccination campaigns and wild poliovirus. To simulate this, we devised a reinfection model where individuals are administered a vaccine-equivalent dose of Sabin 2 poliovirus (10^6^ infectious viruses) at time intervals randomly drawn from a gamma distribution:

|  | $t_{interval}\sim gamma\left( shape\left( x=age \right),scale=1 \right)$ | (21) |
| --- | --- | --- |
|  | $shape(x=age) = \beta\left( 1-exp(-\alpha x \right)) + \gamma$ | (21a) |

The shape of this gamma distribution ensures that the time interval between infection increases with age. We fit our simulation using a PMC-ABC with 1000 particles and four iterations to the previously fit equation of immunity vs age in the household contact population of Matlab, Bangladesh [4]:

|  | $N_{ab,x=age}=Nab\left( 1+\left( 12x-30 \right) \right)^{-0.24}$ | (22) |
| --- | --- | --- |

Distance was defined as the squared difference between simulated immunity values and immunity values estimated using equation 22 for all integer ages between five and 100. Each iteration modified its acceptance threshold such that only the top 30, ten, and five percent of the distance distribution were accepted for the second, third, and fourth iteration. The mean posterior estimates of each parameter were used as our point-estimates. Immunity was consistent with an age-dependent exposure rate, with children inferred to have been re-exposed more frequently than adults.

**Supplemental –** **Calibrating Transmission**

We calibrated two different transmission models: a single parameter ($\beta_{ma}$) complete mixing transmission model and a four parameter ($\beta_{hh}$, $\beta_{bari}$, $\beta_{village}$, $\beta_{intervillage}$) household community transmission transmission model. $\beta$ represents the number of contacts per shedding individual per timestep. When calibrating transmission, parameter estimates were obtained by examining the profile likelihoods of each parameter sampled from a four-dimensional latin hyperspace cube and calibrated to 1) priors for village and intervillage transmission based on Sabin 2 shedding during the enrollment period across all routine immunization trial arms (villages receiving tOPV and bOPV), and 2) the Sabin 2 shedding profile of infants and their household contacts in bOPV following mass mOPV2 immunization.

**Identifying priors for village and intervillage transmission counts from vaccine transmission during enrollment**

**Intervillage**

During enrollment, a small number of subjects in bOPV2 routine immunization villages were positive for Sabin 2 due to transmission from villages assigned to tOPV routine immunization or the community outside Matlab. While exposure could come from anywhere in Bangladesh (and beyond), we assumed all Sabin 2 exposure in the bOPV villages during the routine immunization/enrollment period originated from the a nearby tOPV-assigned village. This allowed us to derive an upper-bound for $\beta_{intervillage}$.

First, we calculated the intervillage transmission rate between tOPV and bOPV villages during the enrollment period. We define the intervillage transmission rate as the number of observed Sabin 2 transmission events per observed susceptible subjects in bOPV villages per number of tOPV vaccinations in tOPV villages.

|  | $\lambda_{inter_{k}}=\frac{n_{shedding_{k}}}{{n_{observed_{k}}}/{n_{tOPV}}}$ | (23) |
| --- | --- | --- |
|  |  |  |

where k refers the type of individual (infant or noninfants), $n_{shedding_{k}}$ the number of individuals of type k shedding Sabin 2, ${n_{shedding}}_{k}$ the number of individuals of type k observed throughout the enrollment period, and $n_{tOPV}$ is the total number of tOPV vaccinations administered.

For infants, we followed 625 infants in the bOPV villages during the enrollment period, of which six shed Sabin 2. Similarly, we followed 1137 noninfants (the household contacts of enrolled individuals), of which one shed Sabin 2.

|  | $\lambda_{inter_{infant}}=\frac{6}{{625}/{600}}=1.6*{10}^{-5}$ | (24) |
| --- | --- | --- |

| $\lambda_{inter_{noninfant}}=\frac{1}{{1137}/{600}}=1.5*{10}^{-6}$ |
| --- |

The estimated ten-fold lower rate to noninfants is consistent with the differences in immunity between infants who did not receive live Sabin 2 vaccination verses older individuals who have.

Using these rates, we then estimated intervillage transmission events after the onset of the mOPV2 campaign. For simplicity, we assumed that mOPV2 challenge provides an equivalent source of virus as tOPV vaccination in unimmunized infants [4]. We also assume that all non-infants in the population have equivalent intervillage exposure as household contacts. The expected number of intervillage transmissions is estimated as

|  | $N_{inter}=\eta\left[ {\lambda_{inter}}_{infant}*n_{infants}+{\lambda_{inter}}_{noninfant}*n_{noninfant} \right]$ | (25) |
| --- | --- | --- |
|  | $\eta={n_{infants}}_{mOPV2}+\frac{{n_{noninfant}}_{mOPV2}}{15}$ |  |
|  |  |  |

Where $N_{inter}$ is the number of intervillage transmission events in bOPV villages post-mOPV2 challenge, $\eta$is the number of infant-equivalent mOPV2 recipients,${n_{infants}}_{mOPV2}$ is the number of infants challenged with mOPV2, ${n_{noninfant}}_{mOPV2}$ is the number of household contacts challenged with mOPV2, and $n_{infant}$,$n_{noninfant}$ are the number of susceptible (not challenged with mOPV2) infants and noninfants. Because household contacts were older and received tOPV as routine immunization prior to our study, they were observed to shed 15x less virus after mOPV2 challenge [4] . $\eta$ normalizes the difference in shedding following mOPV2 challenge in infants who received bOPV2 and noninfants who received tOPV2 during routine immunization.

In the bOPV villages, 199 infants and 2822 noninfants were challenged with mOPV2. The total number of susceptible infants and household contacts was ~1200 and ~80000.

|  | $\eta=199+\frac{2822}{15}\approx387$ |  |
| --- | --- | --- |
|  | $N_{inter}=387\left[ {\lambda_{inter}}_{infant}*1200+{\lambda_{inter}}_{hhc}*80000 \right]\approx54$ | (26) |

Thus, to constrain intervillage transmission in our household community transmission model, we assume a prior for $\beta_{intervillage}$ using a normal distribution with mean $N_{iv}$ and variance 10*$N_{iv}$. The ten serves to inflate variance because $N_{iv}$ was only crudely estimated. The log-likelihood component for intervillage transmission was defined as

|  | $logL_{inter}=-\frac{\left( {N_{inter}}_{simulated}-N_{inter} \right)^{2}}{2*(10*N_{inter})}$ | (27) |
| --- | --- | --- |

**Within-Village**

To constrain the within-village transmission parameter, we examined enrollment data from the tOPV villages. For subjects in the tOPV villages, exposure prior to the first dose of routine immunization at six weeks of age (Table S1 of [4]) is most likely due transmission from older infants in the village shedding Sabin 2 following tOPV routine immunization. Following this logic, we observed one infant and household contact infection due to within-village transmission (Table S5 of [4]) among 294 infants and 547 household contacts. As above, approximately 600 children received tOPV in routine immunization, spread out across 22 villages. Thus, the average number of tOPV vaccine recipients in each village was 600/22 $\approx$27. However, using the average tOPV vaccine recipient count would overestimate the expected within-village transmission rate, due to the heavy skew in village sizes.

The village-size weighted transmission rate was estimated as a weighted sum across all villages

|  | $\lambda_{village_{k}}=\sum_{i}^{n\_villages_{tOPV}} \frac{n_{shedding_{i,k}}}{{{n_{observed}}_{k,i}}/{{n_{tOPV}}_{i}}} \left( \frac{{n_{individuals}}_{i}}{{n_{individuals}}_{total}} \right)$ | (28) |
| --- | --- | --- |

Which yielded:

|  | $\lambda_{village_{infant}}=1*{10}^{-4}$ | (29) |
| --- | --- | --- |
|  | $\lambda_{village_{noninfant}}=7.5*{10}^{-5}$ | (30) |

Similarly, the weighted total number of within village events was defined as

|  | $N_{village}=\eta\left[ {\lambda_{village}}_{infant}*n_{infants}+{\lambda_{village}}_{noninfant}*n_{noninfant} \right]*\sum_{i}^{n\_villages_{tOPV}} \left( \frac{{n_{individuals}}_{i}}{{n_{individuals}}_{total}} \right)$ | (31) |
| --- | --- | --- |
|  | $\eta={n_{infants}}_{mOPV2}+\frac{{n_{noninfant}}_{mOPV2}}{15}$ |  |

We found that $N_{village}=140$. As with intervillage transmission, we assumed a prior for within village transmission events as a normal distribution with mean $N_{village}=140$ and variance ${10*N}_{village}$. The log-likelihood component for within village transmission was defined as:

|  | $logL_{village}=-\frac{\left( {N_{village}}_{simulated}-N_{village} \right)^{2}}{2*(10*N_{village})}$ | (32) |
| --- | --- | --- |

**Why priors and not explicit modeling of the enrollment period?**

The decision to use informative priors to describe the enrollment and routine immunization data as opposed to calibrating transmission by simulating both the routine immunization and post-mOPV2 campaign was done to avoid an explosion in model complexity for little gain in model identifiability beyond that provided by the priors derived from the enrollment data defined above. Accurately simulating the routine immunization phase would have required combining data regarding the timing of routine immunization for each enrolled infant with the demographic model to properly simulate routine immunization and accurately simulate any residual tOPV-derived transmission following the mass mOPV2 campaign. It was possible to do this in Matlab because data from the unique HDSS program [11] can support constructing near-exact *in silico* replicas. However, to support future generality, we decided instead to simulate populations whose average demographic structure resembled Matlab from data available for many countries through the DHS Program (<https://dhsprogram.com/>) and other surveys. Furthermore, given the small number of transmission events to calibrate to, one could still only calibrate coarse parameters describing average within- and between-village transmission – information that could be incorporated more simply and transparently as described above.

**Shedding proportions in household cohorts**

Stool samples were collected from enrolled study participants 0-10, 14, 18, and 22 weeks post-mOPV2 challenge. Shed prevalence was calculated as the proportion of shedding individuals per household cohort. We assumed that shed prevalence followed a binomial distribution at that timepoints were independent. Under these assumptions, our combined likelihood function was defined as

|  | $logL=$  $\sum_{k}^{n_{traces}} \frac{\left\lceil\sum_{i}^{hh_{cohorts}} \left[ p_{i,k}^{n_{shedding_{i,k}}}*\left( 1-p_{i,k} \right)^{\left( n_{total_{i,k}}-n_{shedding_{i,k}} \right)} \right]+logL_{village,k}+logL_{inter,k} \right\rceil}{n_{traces}}$ | (33) |
| --- | --- | --- |
|  |  |  |

Where k is the simulation trace, i refers to the ith household cohort, p is the proportion of shedding in Matlab, $n_{shedding,i}$ is the number of shedding individuals in the ith household cohort from our simulation, and $n_{total,i}$ the total number of simulated individuals for the ith household cohort. Due to the stochastic nature of our model, we calculated the log-likelihood for each of the 30 simulation traces run and used the average log-likelihood [12].

For our household community transmission model, we first sampled 1800 parameter combinations from a four-dimensional latin hyperspace cube where each dimension corresponded to one component of $\beta$ used in the household community transmission model. We then evaluated the profile likelihoods for each dimension [13]. For the household community transmission model, the profile likelihood surfaces for $\beta_{hh}$ and $\beta_{bari}$ were intractable; low values for either of these parameters could be compensated by increasing transmission at any of the other levels, even after including the additional contraints to within- and inter-village transmission observed during the study enrollment period. Despite this, the initial parameter search showed that $\beta_{hh}$ had a maximum negative likelihood estimate at one. It is highly unlikely that individuals could have a $\beta_{hh}$ of zero, which would indicate no contact with household members.

To identify the remaining parameters, we resampled a total of 2000 points from a 4-dimensional latin hyperspace cube. The dimension associated with $\beta_{hh}$ was replaced with one examining fecal-oral dose concentrations and assumed $\beta_{hh}$ = 1. This forces the model to contact one household member in each timestep. Three fecal-oral doses were examined: 1.25e-6g/contact, 2.5e-6g/contact, and 5e-6g/contact. Of these, 1.25e-6g/contact was the optimal choice. The range of values explored for $\beta_{bari}$, $\beta_{village}$, and $\beta_{intervillage}$ranged from [1,45], [1,10], and [1,10], respectively.

A notable effect of constraining $\beta_{hh}$ to 1 was the identifiability of $\beta_{bari}$ and $\beta_{intervillage}$ (**S6 Fig**). The profile likelihood for $\beta_{bari}$ showed that $\beta_{bari}$ ranging from 15 to 25 were favored, with a maximum of 18. The profile likelihood for $\beta_{intervillage}$ was relatively flat between values one and four but falls sharply at values greater than four. The profile likelihood for $\beta_{village}$ rose steadily until four, after which it remained flat before falling at values greater than 10. In light of this complex parameter landscape, we decided to use the following point-estimates for the multiscale transmission model: fecal-oral dose = 1.25e-6g/contact, $\beta_{hh}$=1, $\beta_{bari}$=18, $\beta_{village}$=4 and $\beta_{intervillage}$=2 (**S5 Fig**).

From a practical perspective, this highlights the importance of collecting transmission data from multiple demographic scales, particularly when trying to assess the impact of household community structure on heterogeneous transmission. It is likely that parameter identifiability would be much improved if additional data that directly quantified household or *bari* transmission were obtained.

For our mass action model, we sampled $\beta_{ma}$ from a set of consecutive integer values ranging from 0-30 and evaluated the average log-likelihood at each point. To make it equivalent with the household community transmission model, we assumed fecal-oral dose was 1.25e-6g/contact. We evaluated $\beta_{ma}$ using two different log-likelihood functions, one with the village and inter-village priors (above equation), and one without:

|  | $logL=\sum_{k}^{n_{traces}} \frac{\sum_{i}^{hh_{cohorts}} \left[ p_{i,k}^{n_{shedding_{i,k}}}*\left( 1-p_{i,k} \right)^{\left( n_{total_{i,k}}-n_{shedding_{i,k}} \right)} \right]}{n_{traces}}$ | (34) |
| --- | --- | --- |

With the village and inter-village priors, our point estimate for $\beta_{global}$ = 1. Without the village and inter-village priors, our point-estimate for $\beta_{global}$=19 (**S6 Fig**).

The priors for village and inter-village transmission were instrumental for calibrating both the mass action and multiscale transmission models. Given their importance for parameter identification, we performed a modified sensitivity analysis by modifying the variance of the normal distribution used to describe the priors for village and inter-village transmission (Equation 27 and Equation 32). The variance represents the confidence in the average estimate for within and inter-village transmission and we extended our analyses to examine variances that were 1 (indicating higher confidence), 10 (the previous estimate), and 1000 (indicating low confidence) times the village size. Increasing the variance term flattened the profile likelihoods of $\beta_{village}$ and $\beta_{intervillage}$ but otherwise had little effect on our estimated point estimates. The constraint on within- and inter-village transmission was lost when the variance was 1000 times the village size (**S6 Fig**).

The Akaike Information Criterion (AIC) was used to compare model fits between the multiscale and mass action model.

|  | $AIC=2k-2ln(L)$ | (35) |
| --- | --- | --- |

where k is the number of free parameters to be estimated and *L* is the likelihood of the parameter point estimates used in the study. *k* was set to one for the mass action model and four for the multiscale model. *L* was defined by either equation 33 or equation 34, depending on whether the models were calibrated with (equation 33) or without the village and inter-village priors (equation 34).

**Simulating the mOPV2 challenge and post-mOPV2 surveillance period**

The 45 bOPV-assigned villages were simulated using a two-phase burn-in designed to simulate the decline in fertility and mortality observed in Bangladesh [14]. The tOPV-assigned villages were excluded to avoid simulating any residual, tOPV-derived Sabin 2 transmission. Infant immunity was defined by assigning an OPV-equivalent antibody titer consistent with heterologous bOPV-induced Sabin 2 immunity. Immunity in older individuals was assigned by assuming repeated reinfection from historical vaccination campaigns and was calibrated to the observed shedding rates of the greater Matlab population.

The mOPV2 challenge and subsequent surveillance period was simulated by:

1. Randomly enrolling half the infants in each village to be monitored for Sabin 2 shedding. A third of the enrolled infants received mOPV2, which was simulated using the poliovirus dose response model by assuming a viral exposure dose of 10^6^ CCID50 units.
2. Enrolling the two household contacts of each enrolled infant for stool surveillance. Priority was given to the two youngest individuals < 14 years of age. If not possible, the next youngest female household members were chosen until two household contacts could be obtained, which reflects the sampling used in the study [4]. Five percent of enrolled household contacts were challenged with mOPV2. The enrolled infants and household contacts were divided into eight household cohorts defined in **Table 1**.
3. Challenging 40% of all children < five years of age with mOPV2.
4. Simulating onward transmission assuming complete mixing or household community transmission in single day time steps for 22 weeks. Changes in household community structure are also updated using the household evolution model.
5. Shedding of all enrolled individuals was recorded at weekly intervals for the first 10 weeks, then at weeks 14, 18, and 22. The proportion of shedding individuals in each cohort, at each recorded time point, was then output by the model. Where each successful transmission originated from (from a household, bari, village, or non-vilalge member) was also recorded.

*Extrapolating Sabin 2 vaccine virus and cVDPV2 transmission risk*

When extrapolating Sabin 2 vaccine virus and cVDPV2 transmission risk following the Switch, the populations used to simulate the mOPV2 vaccination campaigns were advanced for a period of up to 40 years. During this period, the population was updated by allowing household community structure to change due to new births and deaths and by allowing pre-existing immunity to wane. Newly born individuals were not vaccinated against Sabin 2 and we assumed all demographic parameters relating to fertility, mortality, and marriage, were the same as those in 2014. cVDPV2 infections were simulated by altering the shedding duration and infectivity parameters of the poliovirus infection model using the values reported in the supplemental parameter table of [3].

**SI References**

1. Skinner GW. Family Systems and Demographic Processes. In: Kertzer DI, Fricke T, editors. Anthropological Demography: Toward a New Synthesis. University of Chicago Press; 1994.

2. Amin S. Family Structure and Change in Rural Bangladesh. Population Studies. 1998;52: 201–213.

3. Famulare M, Selinger C, McCarthy KA, Eckhoff PA, Chabot-Couture G. Assessing the stability of polio eradication after the withdrawal of oral polio vaccine. PLOS Biology. 2018;16: e2002468. Available: https://doi.org/10.1371/journal.pbio.2002468

4. Taniuchi M, Famulare M, Zaman K, Uddin MJ, Upfill-Brown AM, Ahmed T, et al. Community transmission of type 2 poliovirus after cessation of trivalent oral polio vaccine in Bangladesh: an open-label cluster-randomised trial and modelling study. The Lancet Infectious diseases. 2017;17: 1069–1079. doi:10.1016/S1473-3099(17)30358-4

5. Behrend MR, Hu H, Nigmatulina KR, Eckhoff P. A quantitative survey of the literature on poliovirus infection and immunity. International Journal of Infectious Diseases. 2014;18: 4–13. doi:https://doi.org/10.1016/j.ijid.2013.09.005

6. Alexander JPJ, Gary HEJ, Pallansch MA. Duration of poliovirus excretion and its implications for acute flaccid paralysis surveillance: a review of the literature. The Journal of infectious diseases. 1997;175 Suppl: S176-82.

7. O’Ryan M, Bandyopadhyay AS, Villena R, Espinoza M, Novoa J, Weldon WC, et al. Inactivated poliovirus vaccine given alone or in a sequential schedule with bivalent oral poliovirus vaccine in Chilean infants: a randomised, controlled, open-label, phase 4, non-inferiority study. The Lancet Infectious diseases. 2015;15: 1273–1282. doi:10.1016/S1473-3099(15)00219-4

8. Asturias EJ, Bandyopadhyay AS, Self S, Rivera L, Saez-Llorens X, Lopez E, et al. Humoral and intestinal immunity induced by new schedules of bivalent oral poliovirus vaccine and one or two doses of inactivated poliovirus vaccine in Latin American infants: an open-label randomised controlled trial. Lancet (London, England). 2016;388: 158–169. doi:10.1016/S0140-6736(16)00703-0

9. Jafari H, Deshpande JM, Sutter RW, Bahl S, Verma H, Ahmad M, et al. Efficacy of inactivated poliovirus vaccine in India. Science. 2014;345: 922 LP – 925. doi:10.1126/science.1255006

10. GELFAND HM, LEBLANC DR, FOX JP, CONWELL DP. Studies on the development of natural immunity to poliomyelitis in Louisiana. II. Description and analysis of episodes of infection observed in study group households. American journal of hygiene. 1957;65: 367–385.

11. Alam N, Ali T, Razzaque A, Rahman M, Zahirul Haq M, Saha SK, et al. Health and Demographic Surveillance System (HDSS) in Matlab, Bangladesh. International Journal of Epidemiology. 2017;46: 809–816. doi:10.1093/ije/dyx076

12. He D, Ionides EL, King AA. Plug-and-play inference for disease dynamics: measles in large and small populations as a case study. Journal of the Royal Society, Interface. 2009/06/17. 2010;7: 271–283. doi:10.1098/rsif.2009.0151

13. L. IE, C. B, J. P, A. SR, A. KA. Monte Carlo profile confidence intervals for dynamic systems. Journal of The Royal Society Interface. 2017;14: 20170126. doi:10.1098/rsif.2017.0126

14. Bangladesh Demographic and Health Survey 2014. Dhaka, Bangladesh, and Rockville, Maryland, USA;
