# Supplementary figures and images for: Multiscale model for forecasting Sabin 2 vaccine virus household and community transmission
Source: PLoS Comput Biol. 2021 Dec 21;17(12):e1009690. doi: 10.1371/journal.pcbi.1009690 (PMC8726461; doi:10.1371/journal.pcbi.1009690)

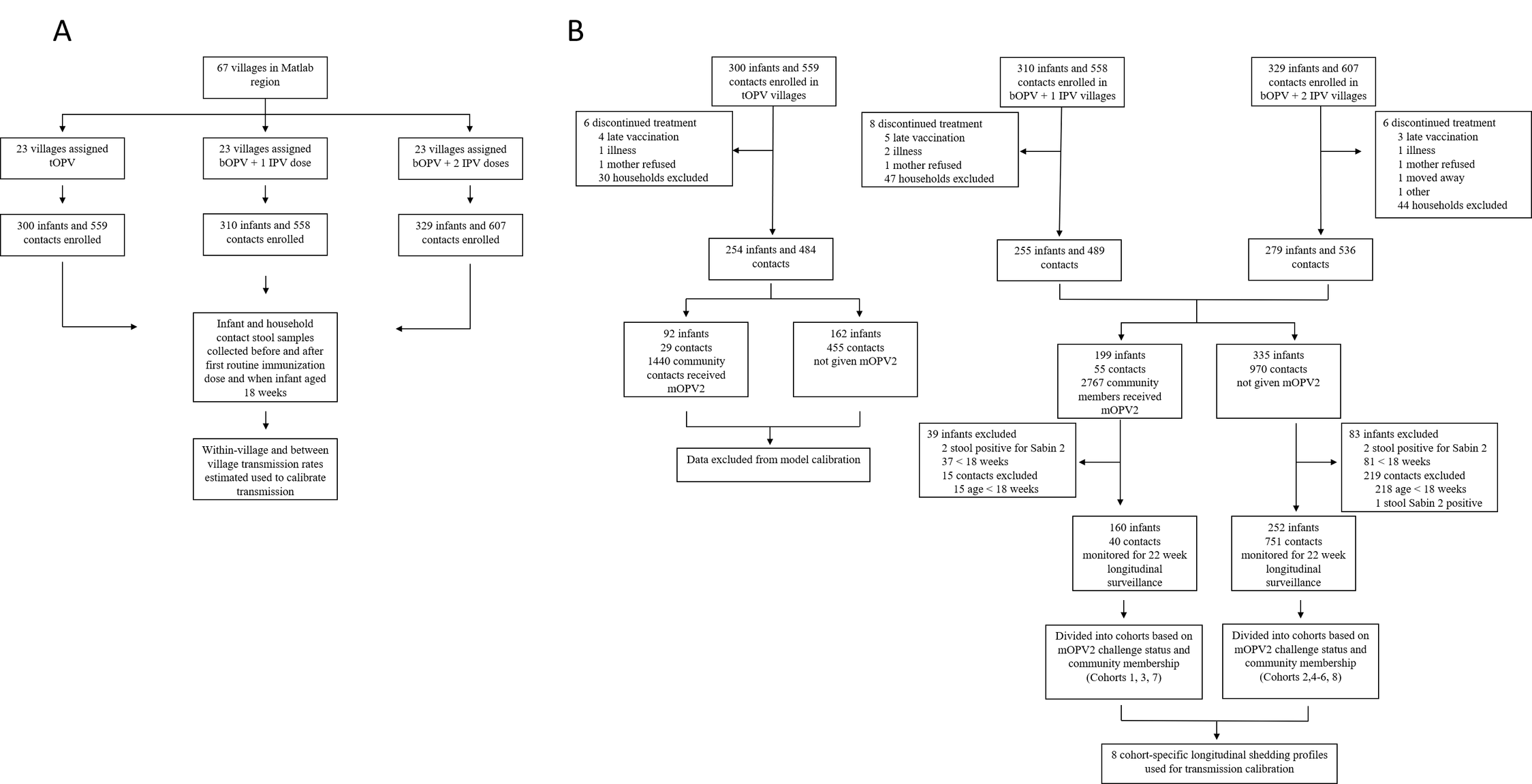

Supplement: S1 Fig — A) Routine immunization and enrollment phase and B) the mOPV2 campaign and the 22 week longitudinal surveillance period. The tOPV villages was excluded from simulation due to the larger potential of unmodeled vaccine transmission from routine immunization. (TIF) [file pcbi.1009690.s003.tif]

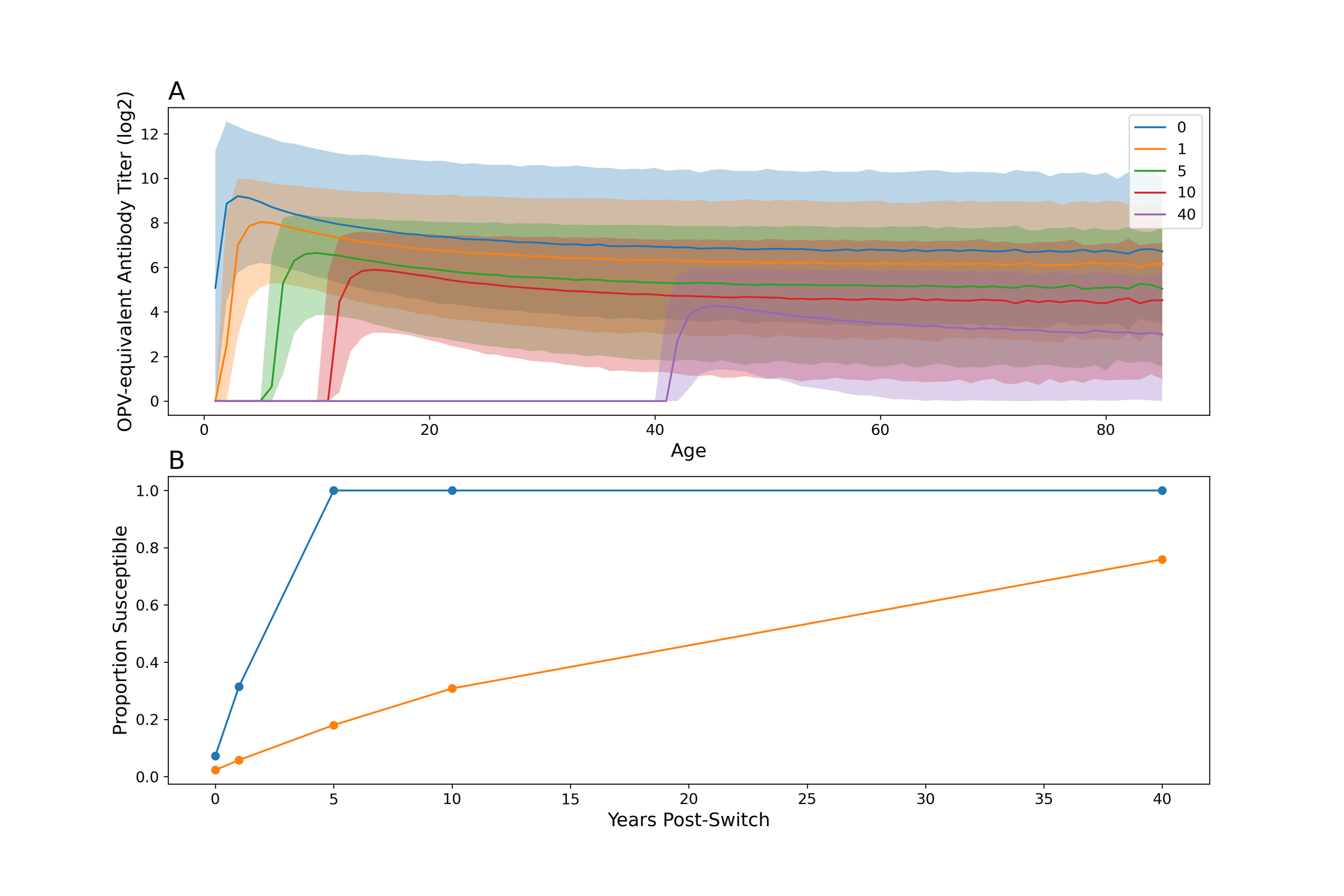

Supplement: S2 Fig — A) Average immunity (log2 OPV-equivalent antibody titers) against Sabin 2 in our populations immediately after (zero years), one, five, ten, and 40 years after the Switch. Solid line indicates the population average and the shading the boundaries of the middle 95th percentile. Note the age-structured erosion of population immunity due to new births and immune waning. B) The proportion of susceptible children under five (blue) and the proportion of susceptible individuals in the population (orange) against years since the Switch. (TIFF) [file pcbi.1009690.s004.tiff]

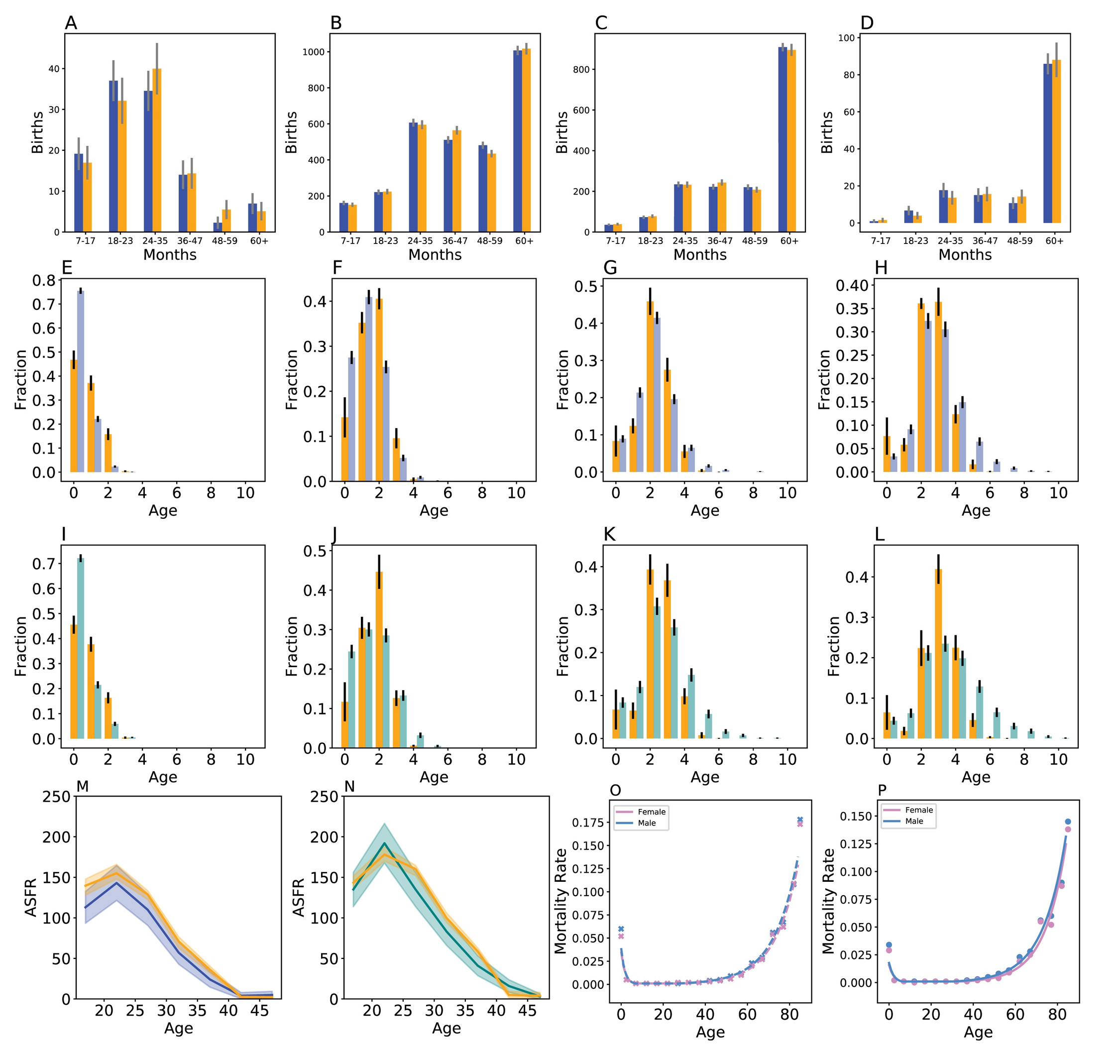

Supplement: S3 Fig — A-D) The birth interval periods for individuals aged A) 15–19, B) 20–29, C) 30–39, and D) 40–49 in 2014. The number of children per married female in 2014 (E-H) and 2004 (I-L) for individuals aged E/I) 15–19, F/J) 20–24, G/K) 25–29, and H/L) 30–34. The age specific fertility rate (ASFR, number of births per 1000 individuals) in 2014 (M) and 2004 (N). For subplots A-N, simulated results are presented in orange, data from 2014 are presented in blue, and data from 2004 are presented in teal. Error bars in all bar plots indicate one binomial standard error from the mean. The shading in plots M and N indicate the middle 95% confidence intervals of the data. Simulated confidence intervals were generated by replicating the simulation 100 times. O-N) The sex-(blue = male, pink = female) and age-specific mortality rates in 2014 (O) and 2004 (P). WHO mortality rates are represented by dots. (TIF) [file pcbi.1009690.s005.tif]

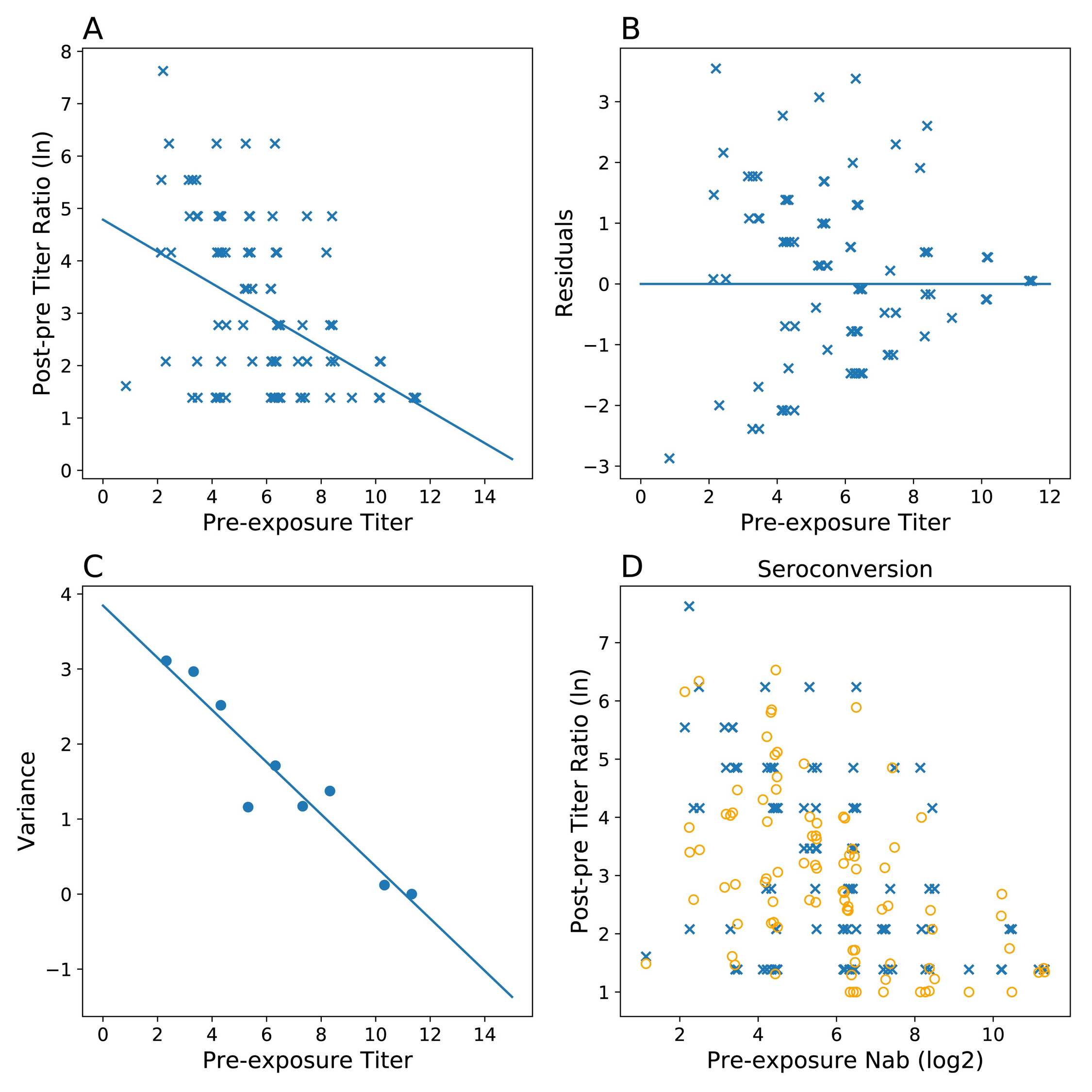

Supplement: S4 Fig — A) Ordinary least squares fit relating pre-exposure log2 antibody titers to the ratio of post-exposure to pre-exposure antibody titer observed in 1953 Louisiana.[37] Overlapping points are randomly jittered to better represent point density. Line represents the ordinary least squares fitted equation. B) Residual plot of our ordinary least squares fit. The fan-shaped distribution is classic signature of heteroskedasticity. C) Fitted ordinary least square function relating variance with pre-exposure titer. D) Final immune boosting model predictions (orange) compared against the original data (blue). (TIF) [file pcbi.1009690.s006.tif]

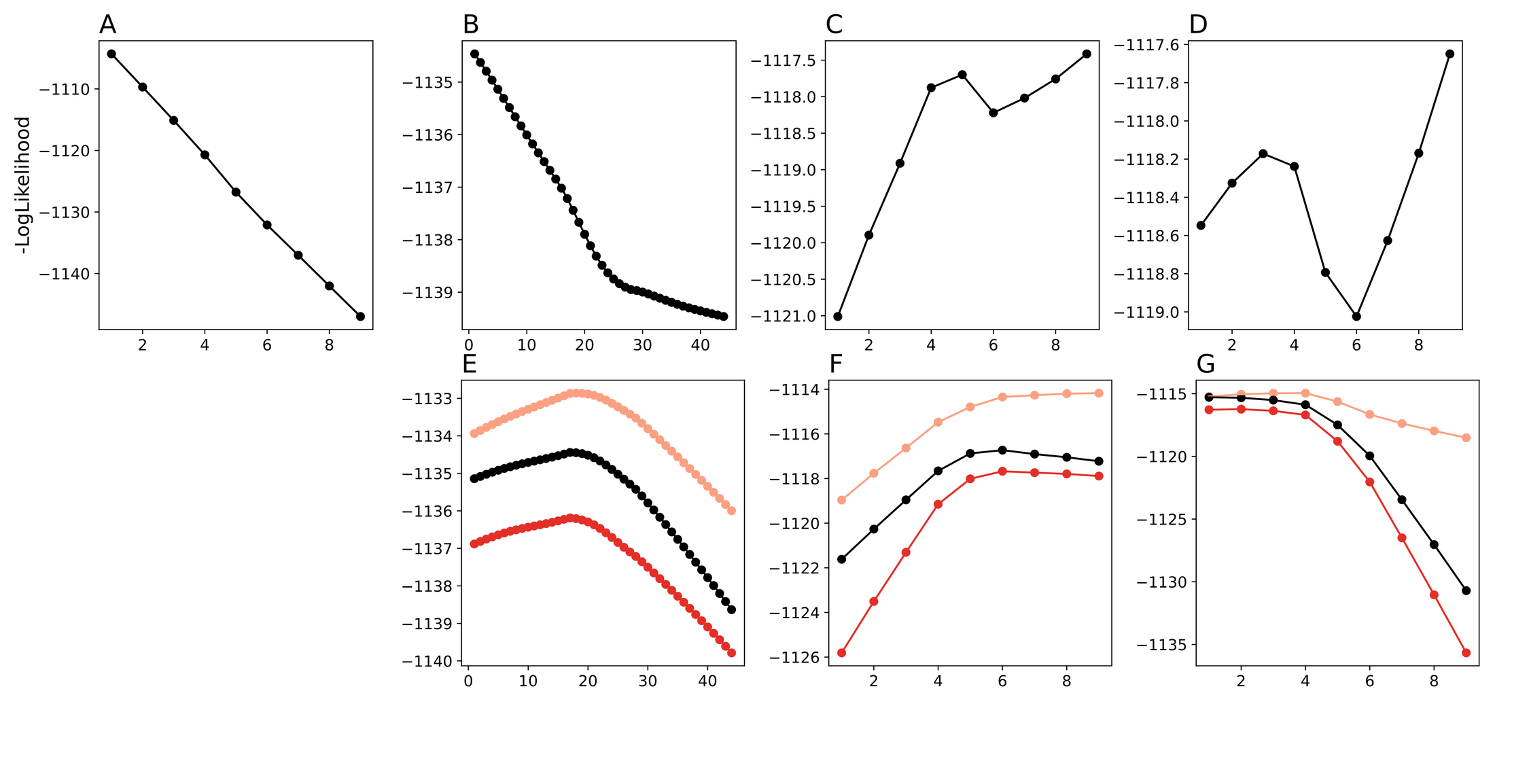

Supplement: S5 Fig — Profile likelihoods of for the four mass action transmission parameters: A) βhh, B/E) βbari, C/F) βvillage, and D/G) βintervillage. A-D were generated without the priors for within- and between- village transmission from the tOPV data. Note the multiple peaks present in D and the non-monotonic decrease in negative log-likelihood in B. E-G were generated with the priors. For E-G, the black profile likelihood represents the profile used to parameterize the multiscale model. The dark and light red likelihoods show the profile likelihoods assuming a higher and lower confidence in the accuracy of the tOPV-derived priors. (TIF) [file pcbi.1009690.s007.tif]

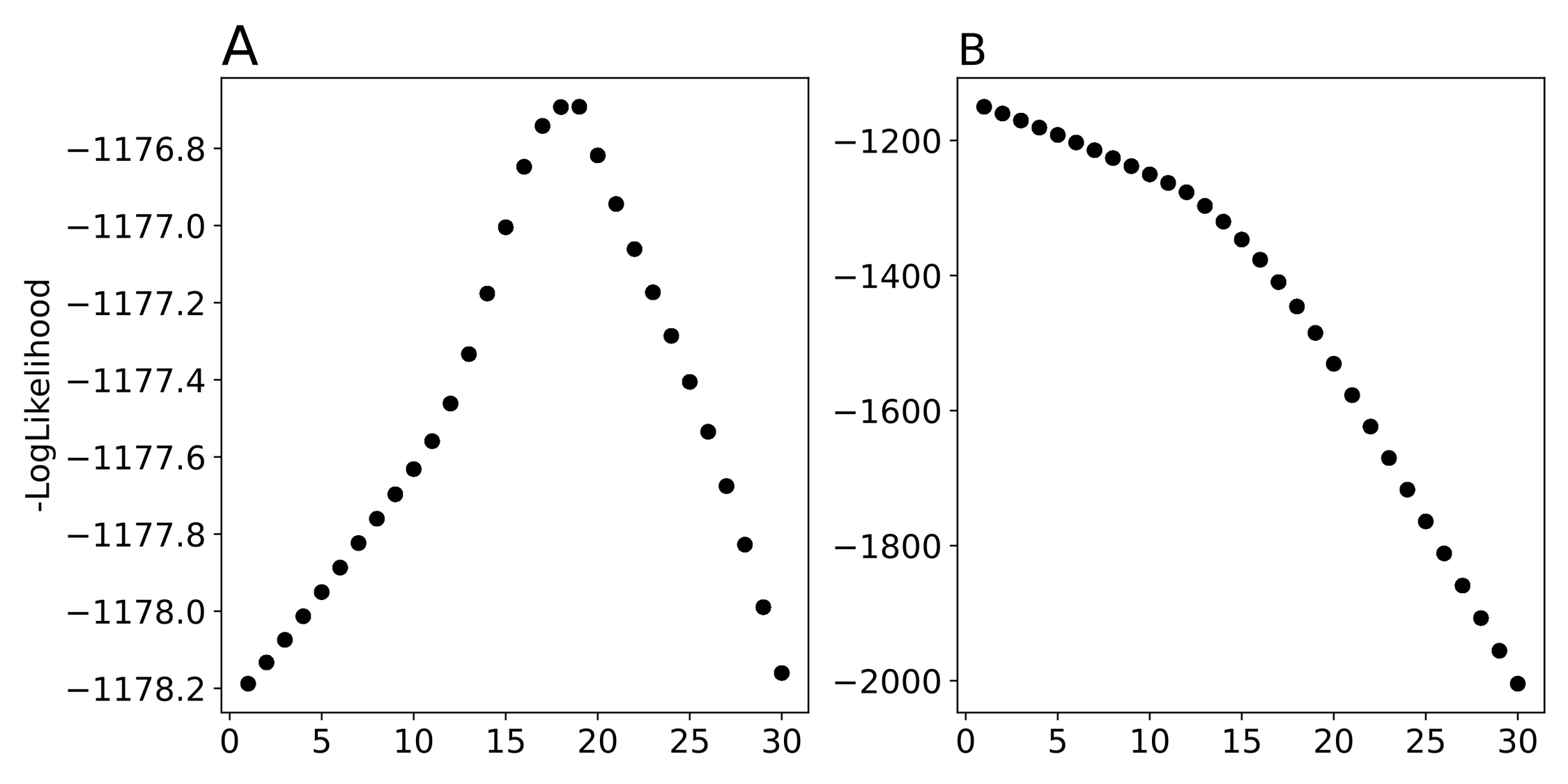

Supplement: S6 Fig — Profile likelihoods for the single-parameter mass action model (βma) without (A) and with (B) the priors for within- and between- village transmission from the tOPV data. (TIF) [file pcbi.1009690.s008.tif]
